# Discovery of indole-3-butyric acid derivatives as potent histone deacetylase inhibitors

**DOI:** 10.1080/14756366.2020.1870457

**Published:** 2021-01-14

**Authors:** Yiming Chen, Lihui Zhang, Lin Zhang, Qixiao Jiang, Lei Zhang

**Affiliations:** a Department of Medicinal Chemistry, School of Pharmacy, Weifang Medical University, Weifang, Shandong, China; b School of Stomatology, Weifang Medical University, Weifang, Shandong, China; c Department of Toxicology, School of Public Health, Qingdao University, Qingdao, Shandong, China

**Keywords:** Histone deacetylase, inhibitor, anticancer, indole

## Abstract

In discovery of HDAC inhibitors (HDACIs) with improved anticancer potency, structural modification was performed on the previous derived indole-3-butyric acid derivative. Among all the synthesised compounds, molecule **I13** exhibited high HDAC inhibitory and antiproliferative potencies in the *in vitro* investigations. The IC_50_ values of **I13** against HDAC1, HDAC3, and HDAC6 were 13.9, 12.1, and 7.71 nM, respectively. In the cancer cell based screening, molecule **I13** showed increased antiproliferative activities in the inhibition of U937, U266, HepG2, A2780, and PNAC-1 cells compared with SAHA. In the HepG2 xenograft model, 50 mg/kg/d of **I13** could inhibit tumour growth in athymic mice compared with 100 mg/kg/d of SAHA. Induction of apoptosis was revealed to play an important role in the anticancer potency of molecule **I13**. Collectively, a HDACI (**I13**) with high anticancer activity was discovered which can be utilised as a lead compound for further HDACI design.

## Introduction

Histone deacetylases (HDACs) are a family of enzymes involved in the deacetylation of histones and non-histone proteins including transcription factors, Hsp90 and tubulin^1–3^. To date, a total of 18 different isoforms of HDACs classified into two families and four classes have been identified in human^4^. The two families are the zinc dependent class I, II, and IV, and the nicotinamide-adenine-dinucleotide (NAD) dependent class III. Class I HDACs contain HDAC1, 2, 3, and 8, and class II HDACs are subdivided into IIa (HDAC4, 5, 7, and 9) and IIb (HDAC6 and 10). Class III HDACs, as a separate family, are a group of NAD dependent silent information regulator 2-related proteins (known as sirtuins, sirt1-7). Class IV HDAC, HDAC11, is structurally different from either class I or class II HDACs.

The acetylation level of histone and non-histone proteins is an opposing activity regulated by HDACs and histone acetyltransferases (HATs)^5–7^. The epigenetic acetylation plays a key role in the regulation of fundamental cellular functions including protein phosphorylation, signal transduction, cell cycle, proliferation, apoptosis, cardiac development, and so on^8–10^. The abnormal expression of HDACs is associated with the pathogenesis of a variety of diseases such as diabetes mellitus^11^, neurodegenerative diseases^12^^,^^13^, inflammatory disorders^14^, HIV^15–17^, cardiac diseases^18^^,^^19^, and especially tumor^20^. Pharmacological inhibition of HDACs and development of HDAC inhibitors (HDACIs) have been widely investigated in the treatment of cancer and other diseases^21^.

HDAC inhibitors have been extensively studied in the treatment of tumour and other epigenetic disorders^20^. According to the chemical structure, HDACIs are divided into groups, including hydroxamic acids, aminobenzamides, cyclic peptides, carboxylic acids, and hybrid molecules^4^. Suberoylanilide hydroxamic acid (SAHA)^22^ and FK228^23^ have been approved by US Food and Drug Administration (FDA) for the treatment of refractory cutaneous T-cell lymphoma (CTCL). PDX101^24^ and LBH589^25^ have been approved for the treatment of peripheral T-cell lymphoma (PTCL) and multiple myeloma, respectively. Chidamide^26^ is a benzamide HDACI approved by Chinese Food and Drug Administration (CFDA) for the treatment of relapsed or refractory PTCL.

**Figure 1.**
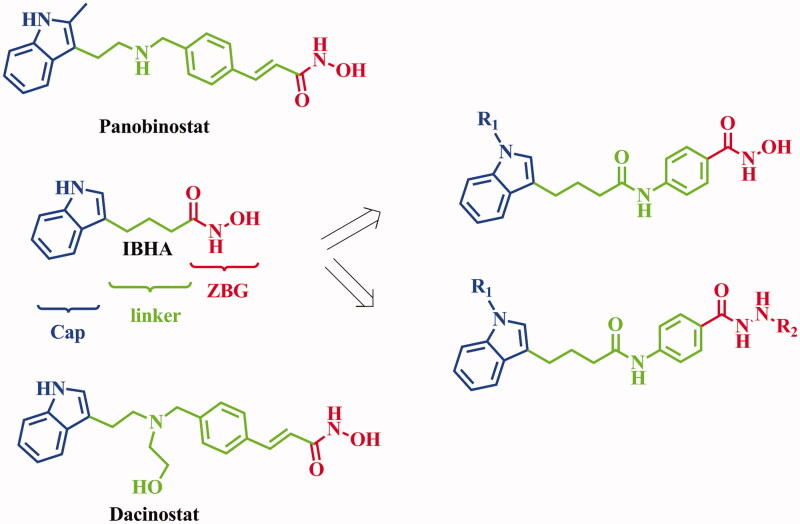
Design of indole-3-butyric acid derivatives from the structure of IBHA. R_1_ group is substituted aromatic rings, R_2_ group is alkanes.

Auxins are a group of plant hormones that play a cardinal role in the plant growth and behavioural processes^27^. Biological effects of naturally occurring auxins in plants include indole-3-acetic acid (IAA), 4-chloroindole-3-acetic acid (4-Cl-IAA), phenylacetic acid (PAA), indole-3-butyric acid (IBA), and indole-3-propionic acid (IPA), have exhibited inhibitory activities against human tumour cell lines^28^. However, development of auxins as anticancer agents has been rarely reported. In our previous study, a potent IBHA with antitumor activity was derived by substitution of carboxyl group of IBA with hydroxamic acid^29^. Compared with SAHA, IBHA exhibited reduced activities in the HDAC enzymatic inhibition and antiproliferative assays. Inspired by structures of high potent HDACIs Panobinostat and Dacinostat which have the 3-alkyl indole pharmacophores in the cap region, structural modification was performed to IBHA. In the present study, to enhance the binding affinity of IBA derivatives with HDACs, and improve the antitumor activity of IBA derivatives, phenyl group was introduced to the linker of IBHA. Substituted groups were introduced to the nitrogen in the indole ring, and hydroxamic acid was utilised as zinc binding group (ZBG). Hydrazide has been reported as ZBG with good HDAC inhibitory activity^30^. Therefore, to investigate the performance of hydrazide in the present structure, hydroxamic acid groups of several active molecules have been replaced by hydrazide groups (Figure 1). The anticancer activities of the derived molecules were evaluated in the enzymatic assay, *in vitro* cancer cell based screening, apoptosis, and *in vivo* anticancer test.

## Materials and methods

All commercially available starting materials, reagents and solvents were used without further purification. All reactions were monitored by TLC with 0.25 mm silica gel plates (60GF-254). UV light and ferric chloride were used to visualise the spots. ^1^H NMR and ^13^C NMR spectra were recorded on a Bruker DRX spectrometer at 500 MHz, using TMS as an internal standard. High-resolution mass spectra were performed in Weifang Medical University.

Methyl 4-aminobenzoate hydrochloric acid has been synthesised and described in our previous work.

Preparation of **I2a** and its analogues: Derivatives **I3a**–**I13a** were prepared as described for **I2a** (see below).

4-(1-Benzyl-1H-indol-3-yl)-butyric acid (**I2a**). To a solution of NaH in DMF, were stirred under Ar_2_ and 0 °C. After 30 min, the benzyl bromide was slowly added. The reaction solution was stirred at room temperature for 2 h. Then, the solvent was evaporated under vacuum. The residue was acidified with saturated citric acid, and then extracted with EtOAc (3 × 20 ml). The organic layer was combined, washed with brine (3 × 20 ml) dried over MgSO_4_, and evaporated under vacuum. The desired compound **I2a** was derived by crystallisation in EtOAc as powder. HRMS *m/z*: 292.1338 [M–H]^–^. ^1^H NMR (400 MHz, DMSO) *δ* 12.03 (s, 1H), 7.53 (d, *J* = 7.6 Hz, 1H), 7.38 (d, *J* = 8.2 Hz, 1H), 7.31–7.27 (m, 3H), 7.23 (d, *J* = 7.2 Hz, 1H), 7.21–7.17 (m, 2H), 7.09–7.05 (m, 1H), 7.00–6.97 (m, 1H), 5.35 (s, 2H), 2.70 (t, *J* = 7.6 Hz, 2H), 2.27 (t, *J* = 7.2 Hz, 2H), 1.91–1.83 (m, 2H).

4-(1-Ethyl-1H-indol-3-yl)-butyric acid (**I3a**). HRMS *m/z*: 230.11719 [M–H]^–^. ^1^H NMR (400 MHz, DMSO) *δ* 12.04 (s, 1H), 7.51 (d, *J* = 7.8 Hz, 1H), 7.40 (d, *J* = 8.2 Hz, 1H), 7.16 (s, 1H), 7.12–7.09 (m, 1H), 7.00–6.97 (m, 1H), 4.14 (q, *J* = 7.2 Hz, 2H), 2.68 (t, *J* = 7.6 Hz, 2H), 2.26 (t, *J* = 7.2 Hz, 2H), 1.89–1.82 (m, 2H), 1.33 (t, *J* = 7.2 Hz, 3H).

4-(1-(4-Trifluoromethyl-benzyl)-1H-indol-3-yl)-butyric acid (**I4a**). HRMS *m/z*: 360.12186 [M–H]^–^. ^1^H NMR (400 MHz, DMSO) *δ* 12.06 (s, 1H), 7.67 (d, *J* = 8.2 Hz, 2H), 7.55 (d, *J* = 7.8 Hz, 1H), 7.38–7.31 (m, 4H), 7.11–7.07 (m, 2H), 7.03–6.99 (m, 1H), 5.48 (s, 2H), 2.72 (t, *J* = 7.6 Hz, 2H), 2.29 (t, *J* = 7.2 Hz, 2H), 1.92–1.85 (m, 2H).

4-(1-(2-Trifluoromethyl-benzyl)-1H-indol-3-yl)-butyric acid (**I5a**). HRMS *m/z*: 360.12039 [M–H]^–^. ^1^H NMR (400 MHz, DMSO) *δ* 12.04 (s, 1H), 7.79 (d, *J* = 7.8 Hz, 1H), 7.60 (d, *J* = 7.6 Hz, 1H), 7.47 (m, 2H), 7.26 (s, 1H), 7.17 (d, *J* = 8.0 Hz, 1H), 7.10–7.02 (m, 2H), 6.50 (d, *J* = 6.9 Hz, 1H), 5.58 (s, 2H), 2.75 (t, *J* = 7.6 Hz, 2H), 2.30 (t, *J* = 7.4 Hz, 2H), 1.94–1.86 (m, 2H).

4-(1-(3,4-Difluoro-benzyl)-1H-indol-3-yl)-butyric acid (**I6a**). HRMS *m/z*: 328.11359 [M–H]^–^. ^1^H NMR (400 MHz, DMSO) *δ* 12.10 (s, 1H), 7.53 (d, *J* = 7.8 Hz, 1H), 7.41 (d, *J* = 8.2 Hz, 1H), 7.35 (dd, *J* = 19.2, 8.4 Hz, 1H), 7.32–7.26 (m, 2H), 7.11–7.08 (m, 1H), 7.02–6.99 (m, 2H), 5.35 (s, 2H), 2.70 (t, *J* = 7.6 Hz, 2H), 2.27 (t, *J* = 7.2 Hz, 2H), 1.91–1.83 (m, 2H).

4-(1-(3-Fluoro-benzyl)-1H-indol-3-yl)-butyric acid (**I7a**). HRMS *m/z*: 310.12384 [M–H]^–^. ^1^H NMR (400 MHz, DMSO) *δ* 12.05 (s, 1H), 7.54 (d, *J* = 7.8 Hz, 1H), 7.39 (d, *J* = 8.2 Hz, 1H), 7.33 (dd, *J* = 14.4, 8.0 Hz, 1H), 7.30 (s, 1H), 7.11–6.97 (m, 5H), 5.38 (s, 2H), 2.71 (t, *J* = 7.5 Hz, 2H), 2.28 (t, *J* = 7.4 Hz, 2H), 1.92–1.84 (m, 2H).

4-(1-(3-Bromo-benzyl)-1H-indol-3-yl)-butyric acid (**I8a**). HRMS *m/z*: 370.04352 [M–H]^–^. ^1^H NMR (400 MHz, DMSO) *δ* 12.07 (s, 1H), 7.54 (d, *J* = 7.8 Hz, 1H), 7.44–7.39 (m, 3H), 7.30 (s, 1H), 7.27–7.24 (m, 1H), 7.15 (d, *J* = 7.8 Hz, 1H), 7.11–7.07 (m, 1H), 7.03–6.99 (m, 1H), 5.37 (s, 2H), 2.71 (t, *J* = 7.5 Hz, 2H), 2.28 (t, *J* = 7.4 Hz, 2H), 1.91–1.84 (m, 2H).

4-(1-(3-Chloro-benzyl)-1H-indol-3-yl)-butyric acid (**I9a**). HRMS *m/z*: 326.09418 [M–H]^–^. ^1^H NMR (400 MHz, DMSO) *δ* 12.08 (s, 1H), 7.54 (d, *J* = 7.8 Hz, 1H), 7.40 (d, *J* = 8.2 Hz, 1H), 7.34–7.28 (m, 3H), 7.23 (s, 1H), 7.13–7.07 (m, 2H), 7.03–6.99 (m, 2H), 5.38 (s, 2H), 2.71 (t, *J* = 7.5 Hz, 2H), 2.28 (t, *J* = 7.4 Hz, 2H), 1.91–1.84 (m, 2H).

4-(1-(2,6-Difluoro-benzyl)-1H-indol-3-yl)-butyric acid (**I10a**). HRMS *m/z*: 328.11423 [M–H]^–^. ^1^H NMR (400 MHz, DMSO) *δ* 12.02 (s, 1H), 7.51 (d, *J* = 7.8 Hz, 1H), 7.46–7.39 (m, 2H), 7.17–7.11 (m, 4H), 7.02–6.99 (m, 1H), 7.11–7.07 (m, 1H), 5.37 (s, 2H), 2.66 (t, *J* = 7.6 Hz, 2H), 2.25 (t, *J* = 7.4 Hz, 2H), 1.86–1.79 (m, 2H).

4-(1-(3,5-Bis-trifluoromethyl-benzyl)-1H-indol-3-yl)-butyric acid (**I11a**). HRMS *m/z*: 428.10791 [M–H]^–^. ^1^H NMR (400 MHz, DMSO) *δ* 12.03 (s, 1H), 8.00 (s, 1H), 7.85 (s, 2H), 7.56 (d, *J* = 7.8 Hz, 1H), 7.48 (d, *J* = 8.2 Hz, 1H), 7.40 (s, 2H), 7.13–7.10 (m, 1H), 7.05–7.01 (m, 1H), 5.59 (s, 2H), 2.73 (t, *J* = 7.4 Hz, 2H), 2.26 (t, *J* = 7.4 Hz, 2H), 1.91–1.84 (m, 2H).

4-(1-Butyl-1H-indol-3-yl)-butyric acid (**I12a**). HRMS *m/z*: 258.14868 [M–H]^–^. ^1^H NMR (400 MHz, DMSO) *δ* 12.06 (s, 1H), 7.51 (d, *J* = 7.8 Hz, 1H), 7.40 (d, *J* = 8.2 Hz, 1H), 7.13–7.09 (m, 1H), 7.00–6.97 (m, 1H), 4.01 (q, *J* = 7.0 Hz, 2H), 2.69 (t, *J* = 7.4 Hz, 2H), 2.26 (t, *J* = 7.4 Hz, 2H), 1.90–1.82 (m, 2H), 1.74–1.66 (m, 2H), 1.28–1.18 (m, 2H), 0.87(t, *J* = 7.4 Hz, 3H).

4-(1-methyl-1H-indol-3-yl)-butyric acid (**I13a**). HRMS *m/z*: 216.10153 [M–H]^–^. ^1^H NMR (400 MHz, DMSO) *δ* 12.03 (s, 1H), 7.51 (d, *J* = 7.9 Hz, 1H), 7.36 (d, *J* = 8.2 Hz, 1H), 7.14–7.09 (m, 2H), 7.02–6.98 (m, 1H), 3.72 (s, 3H), 2.68 (t, *J* = 7.5 Hz, 2H), 2.26 (t, *J* = 7.2 Hz, 2H), 1.89–1.81 (m, 2H).

Preparation of **I1b** and its analogues: derivatives **I2b–I13b** were prepared as described for **I1b** (see below).

Methyl 4-(4-(1H-indol-3-yl)butanamido)benzoate (**I1b**). To a solution of IBA (a) (1.0 g, 5 mmol) in DCM (50 ml), Et_3_N (0.55 g, 5.5 mmol) and TBTU (1.8 g, 5.5 mmol) were added in turn. After 20 min, methyl 4-aminobenzoate hydrochloride (0.94 g, 5 mmol) and Et_3_N (0.50 g, 5 mmol) were added. The reaction solution was stirred at room temperature for 8 h. Then, the solvent was evaporated with the residue being taken up in EtOAc (50 ml). The EtOAc solution was washed with saturated citric acid (3 × 20 ml), NaHCO_3_ (3 × 20 ml), and brine (3 × 20 ml), dried over MgSO_4_, and evaporated under vacuum. The desired compound **I1b** (1.2 g, 72% yield) was derived by crystallisation in EtOAc as white powder. HRMS *m/z*: 335.13815 [M + H]^–^. ^1^H NMR (400 MHz, DMSO) *δ* 10.78 (s, 1H), 10.23 (s, 1H), 7.90 (d, *J* = 8.6 Hz, 2H), 7.73 (d, *J* = 8.7 Hz, 2H), 7.52 (d, *J* = 7.8 Hz, 1H), 7.33 (d, *J* = 8.1 Hz, 1H), 7.13 (s, 1H), 7.07–7.04 (m, 1H), 6.98–6.94 (m, 1H), 2.74 (t, *J* = 7.4 Hz, 2H), 2.41 (t, *J* = 7.4 Hz, 2H), 2.01–1.94 (m, 2H).

Methyl 4-(4-(1-benzyl-1H-indol-3-yl)butanamido)benzoate (**I2b**). HRMS *m/z*: 427.20010 [M + H]^+^. ^1^H NMR (400 MHz, DMSO) *δ* 10.24 (s, 1H), 7.90 (d, *J* = 8.6 Hz, 2H), 7.74 (d, *J* = 8.7 Hz, 2H), 7.55 (d, *J* = 7.8 Hz, 1H), 7.39 (d, *J* = 7.8 Hz, 1H), 7.30–7.27 (m, 1H), 7.23 (d, *J* = 7.1 Hz, 1H), 7.18 (d, *J* = 7.1 Hz, 2H), 7.10–7.06 (m, 1H), 7.01–6.98 (m, 1H), 5.35 (s, 2H), 3.81 (s, 3H), 2.75 (t, *J* = 7.4 Hz, 2H), 2.43 (t, *J* = 7.4 Hz, 2H), 2.02–1.95 (m, 2H).

Methyl 4-(4-(1-ethyl-1H-indol-3-yl)butanamido)benzoate (**I3b**). HRMS *m/z*: 365.18481 [M + H]^+^. ^1^H NMR (400 MHz, DMSO) *δ* 10.23 (s, 1H), 7.90 (d, *J* = 8.6 Hz, 2H), 7.73 (d, *J* = 8.7 Hz, 2H), 7.53 (d, *J* = 7.9 Hz, 1H), 7.40 (d, *J* = 8.2 Hz, 1H), 7.18 (s, 1H), 7.13–7.09 (m, 1H), 7.00–6.97 (m, 1H), 4.14 (q, *J* = 7.2 Hz, 2H), 3.81 (s, 3H), 2.73 (t, *J* = 7.4 Hz, 2H), 2.42 (t, *J* = 7.4 Hz, 2H), 2.01–1.93 (m, 2H), 1.33 (t, *J* = 7.2 Hz, 3H).

Methyl 4-(4-(1-(4-trifluoromethyl-benzyl)-1H-indol-3-yl)butanamido)benzoate (**I4b**). HRMS *m/z*: 495.18729 [M + H]^+^. *δ* 10.24 (s, 1H), 7.90 (d, *J* = 8.6 Hz, 2H), 7.73 (d, *J* = 8.7 Hz, 2H), 7.66 (d, *J* = 7.9 Hz, 2H), 7.57 (d, *J* = 8.2 Hz, 1H), 7.38–7.32 (m, 4H), 7.11–7.07 (m, 1H), 7.03–6.99 (m, 1H), 5.48 (s, 2H), 3.81 (s, 3H), 2.76 (t, *J* = 7.4 Hz, 2H), 2.44 (t, *J* = 7.4 Hz, 2H), 2.03–1.96 (m, 2H).

Methyl 4-(4-(1-(2-trifluoromethyl-benzyl)-1H-indol-3-yl)butanamido)benzoate (**I5b**). HRMS *m/z*: 495.18607 [M + H]^+^. *δ* 10.24 (s, 1H), 7.90 (d, *J* = 8.7 Hz, 2H), 7.79 (d, *J* = 6.9 Hz, 1H), 7.73 (d, *J* = 8.7 Hz, 2H), 7.62 (d, *J* = 7.7 Hz, 1H), 7.51–7.44 (m, 2H), 7.28 (s, 1H), 7.17 (d, *J* = 7.9 Hz, 1H), 7.11–7.02 (m, 2H), 6.51 (d, *J* = 7.1 Hz, 1H), 5.57 (s, 2H), 3.81 (s, 3H), 2.79 (t, *J* = 7.2 Hz, 2H), 2.45 (t, *J* = 7.4 Hz, 2H), 2.04–1.97(m, 2H).

Methyl 4-(4-(1-(3,4-difluoro-benzyl)-1H-indol-3-yl)butanamido)benzoate (**I6b**). HRMS *m/z*: 463.18167 [M + H]^+^. *δ* 10.24 (s, 1H), 7.90 (d, *J* = 8.6 Hz, 2H), 7.73 (d, *J* = 8.6 Hz, 2H), 7.55 (d, *J* = 7.7 Hz, 1H), 7.41 (d, *J* = 8.1 Hz, 1H), 7.39–7.23 (m, 3H), 7.12–7.08 (m, 1H), 7.03–6.99 (m, 2H), 5.35 (s, 2H), 3.81 (s, 3H), 2.75 (t, *J* = 7.6 Hz, 2H), 2.43 (t, *J* = 7.2 Hz, 2H), 2.02–1.95(m, 2H).

Methyl 4-(4-(1-(3-fluoro-benzyl)-1H-indol-3-yl)butanamido)benzoate (**I7b**). HRMS *m/z*: 445.19061 [M + H]^+^. *δ* 10.24 (s, 1H), 7.90 (d, *J* = 8.7 Hz, 2H), 7.74 (d, *J* = 8.7 Hz, 2H), 7.56 (d, *J* = 7.8 Hz, 1H), 7.40 (d, *J* = 8.1 Hz, 1H), 7.36–7.31 (m, 2H), 7.11–6.99 (m, 5H), 5.38 (s, 2H), 3.81 (s, 3H), 2.75 (t, *J* = 7.5 Hz, 2H), 2.43 (t, *J* = 7.2 Hz, 2H), 2.02–1.95(m, 2H).

Methyl 4-(4-(1-(3-bromo-benzyl)-1H-indol-3-yl)butanamido)benzoate (**I8b**). HRMS *m/z*: 505.11108 [M + H]^+^. *δ* 10.24 (s, 1H), 7.89 (d, *J* = 8.6 Hz, 2H), 7.73 (d, *J* = 8.7 Hz, 2H), 7.56 (d, *J* = 7.8 Hz, 1H), 7.44–7.41 (m, 3H), 7.33 (s, 1H), 7.28–7.24 (m, 1H), 7.15 (t, *J* = 7.8 Hz, 1H), 7.12–7.08 (m, 1H), 7.03–6.99 (m, 1H), 5.37 (s, 2H), 3.81 (s, 3H), 2.75 (t, *J* = 7.5 Hz, 2H), 2.43 (t, *J* = 7.4 Hz, 2H), 2.02–1.97(m, 2H).

Methyl 4-(4-(1-(3-chloro-benzyl)-1H-indol-3-yl)butanamido)benzoate (**I9b**). HRMS *m/z*: 461.16150 [M + H]^+^. *δ* 10.25 (s, 1H), 7.90 (d, *J* = 8.6 Hz, 2H), 7.73 (d, *J* = 8.7 Hz, 2H), 7.56 (d, *J* = 7.7 Hz, 1H), 7.40 (d, *J* = 8.2 Hz, 1H), 7.34–7.28 (m, 3H), 7.25 (s, 1H), 7.13–7.08 (m, 2H), 7.03–6.99 (m, 1H), 5.38 (s, 2H), 3.81 (s, 3H), 2.76 (t, *J* = 7.4 Hz, 2H), 2.43 (t, *J* = 7.3 Hz, 2H), 2.63–1.95(m, 2H).

Methyl 4-(4-(1-(2,6-difluoro-benzyl)-1H-indol-3-yl)butanamido)benzoate (**I10b**). HRMS *m/z*: 463.18112 [M + H]^+^. *δ* 10.23 (s, 1H), 7.90 (d, *J* = 8.6 Hz, 2H), 7.73 (d, *J* = 8.7 Hz, 2H), 7.53 (d, *J* = 7.9 Hz, 1H), 7.47–7.41 (m, 2H), 7.16–7.12 (m, 4H), 7.03–6.99 (m, 1H), 5.39 (s, 2H), 3.81 (s, 3H), 2.71 (t, *J* = 7.5 Hz, 2H), 2.40 (t, *J* = 7.4 Hz, 2H), 1.99–1.91(m, 2H).

Methyl 4-(4-(1-(3,5-bis-trifluoromethyl-benzyl)-1H-indol-3-yl)butanamido)benzoate (**I11b**). HRMS *m/z*: 563.17468 [M + H]^+^. *δ* 10.24 (s, 1H), 8.01 (s, 1H), 7.91–7.87 (m, 4H), 7.73 (d, *J* = 8.5 Hz, 2H), 7.58 (d, *J* = 7.8 Hz, 1H), 7.48 (d, *J* = 8.0 Hz, 1H), 7.42 (s, 1H), 7.14–7.10 (m, 1H), 7.05–7.01 (m, 1H), 5.58 (s, 2H), 3.82 (s, 3H), 2.76 (t, *J* = 7.4 Hz, 2H), 2.43 (t, *J* = 7.4 Hz, 2H), 2.02–1.95(m, 2H).

Methyl 4-(4-(1-butyl-1H-indol-3-yl)butanamido)benzoate (**I12b**). HRMS *m/z*: 393.21597 [M + H]^+^. *δ* 10.23 (s, 1H), 7.89 (d, *J* = 8.6 Hz, 2H), 7.73 (d, *J* = 8.6 Hz, 2H), 7.53 (d, *J* = 7.8 Hz, 1H), 7.40 (d, *J* = 8.2 Hz, 1H), 7.16 (s, 1H), 7.12–7.08 (m, 1H), 7.00–6.97 (m, 1H), 4.09 (t, *J* = 7.0 Hz, 2H), 3.81 (s, 3H), 2.73 (t, *J* = 7.4 Hz, 2H), 2.41 (t, *J* = 7.2 Hz, 2H), 2.01–1.93(m, 2H), 1.74–1.66(m, 2H), 1.28–1.91 (m, 2H), 0.87 (t, *J* = 7.4 Hz, 3H).

Methyl 4-(4-(1-methyl-1H-indol-3-yl)butanamido)benzoate (**I13b**). HRMS *m/z*: 351.16901 [M + H]^+^. *δ* 10.23 (s, 1H), 7.90 (d, *J* = 8.6 Hz, 2H), 7.73 (d, *J* = 8.7 Hz, 2H), 7.53 (d, *J* = 7.9 Hz, 1H), 7.37 (d, *J* = 8.2 Hz, 1H), 7.14–7.12 (m, 2H), 7.02–6.98 (m, 1H), 3.81 (s, 3H), 3.72 (s, 3H), 2.73 (t, *J* = 7.3 Hz, 2H), 2.41 (t, *J* = 7.4 Hz, 2H), 2.00–1.93(m, 2H).

Preparation of **I1** and its analogues: derivatives **I2–I13** were prepared as described for **I1** (see below).

4-(4-(1H-indol-3-yl)butanamido)-N-hydroxybenzamide (**I1**). Compound methyl 4-(4-(1H-indol-3-yl)butanamido)benzoate (I1a) (0.34 g, 1.0 mmol) was dissolved in 14 ml of NH_2_OK (0.56 g, 24 mmol) methanol solution. After 2 h, the solvent was evaporated under vacuum. The residue was acidified with saturated citric acid, and then extracted with EtOAc (3 × 20 ml). The organic layers were combined, washed with brine (3 × 20 ml) and dried over MgSO_4_. The desired compound **c** (0.16 g, 46% yield) was derived by crystallisation in EtOAc as white powder. HRMS (AP-ESI) *m/z* calcd for C_19_H_20_N_3_O_3_ [M + H]^+^ 338.14600, found 338.34195. ^1^H NMR (400 MHz, DMSO) *δ* 11.08 (s, 1H), 10.78 (s, 1H), 10.09 (s, 1H), 8.93 (s, 1H), 7.68 (dd, *J* = 18.4, 8.8 Hz, 4H), 7.53 (d, *J* = 7.8 Hz, 1H), 7.34 (d, *J* = 8.0 Hz, 1H), 7.14 (s, 1H), 7.08–7.04 (m, 1H), 6.99–6.95 (m, 1H), 2.74 (t, *J* = 7.36 Hz, 2H), 2.40 (t, *J* = 7.4 Hz, 2H), 2.01–1.94 (m, 2H); ^13^C NMR (400 MHz, DMSO): *δ* = 172.14, 164.46, 142.34, 136.79, 128.12, 127.63, 127.38, 122.80, 121.32, 118.77, 118.61, 114.42, 111.81, 36.66, 26.25, 24.73 ppm.

4-(4-(1-benzyl-1H-indol-3-yl)butanamido)-N-hydroxybenzamide (**I2**). HRMS (AP-ESI) *m/z* calcd for C_26_H_25_N_3_O_3_ [M + H]^+^ 428.19295, found 428.19577. ^1^H NMR (400 MHz, DMSO) *δ* 11.08 (s, 1H), 10.10 (s, 1H), 8.92 (s, 1H), 7.67 (dd, *J* = 18.4, 8.8 Hz, 4H), 7.54 (d, *J* = 7.8 Hz, 1H), 7.39 (d, *J* = 8.4 Hz, 1H), 7.30–7.27 (m, 3H), 7.23 (d, *J* = 7.0 Hz, 1H), 7.18 (d, *J* = 7.4 Hz, 2H), 7.10–7.06 (m, 1H), 7.01–6.98 (m, 1H), 5.35 (s, 2H), 2.75 (t, *J* = 7.2 Hz, 2H), 2.41 (t, *J* = 7.5 Hz, 2H), 2.01–1.96 (m, 2H); ^13^C NMR (400 MHz, DMSO): *δ* = 172.07, 164.44, 142.38, 138.91, 136.58, 128.96, 128.26, 128.12, 127.71, 127.47, 127.39, 126.73, 121.64, 119.20, 118.94, 118.75, 114.51, 110.49, 49.37, 36.68, 26.24, 24.65 ppm.

4-(4-(1-Ethyl-1H-indol-3-yl)butanamido)-N-hydroxybenzamide (**I3**). HRMS (AP-ESI) *m/z* calcd for C_21_H_23_N_3_O_3_ [M + H]^+^ 366.17730, found 366.18027. ^1^H NMR (400 MHz, DMSO) *δ* 11.08 (s, 1H), 10.09 (s, 1H), 8.92 (s, 1H), 7.67 (dd, *J* = 19.0, 8.7 Hz, 4H), 7.53 (d, *J* = 7.9 Hz, 1H), 7.40 (d, *J* = 8.3 Hz, 1H), 7.18 (s, 1H), 7.13–7.09 (m, 1H), 7.01–6.97 (m, 1H), 4.14 (q, *J* = 7.2 Hz, 2H), 2.73 (t, *J* = 7.4 Hz, 2H), 2.40 (t, *J* = 7.4 Hz, 2H), 1.98–1.93 (m, 2H), 1.33 (t, *J* = 7.2 Hz, 3H); ^13^C NMR (400 MHz, DMSO): *δ* = 172.05, 164.40, 142.35, 136.16, 128.12, 128.07, 127.38, 125.59, 121.38, 119.14, 118.72, 118.66, 113.96, 110.00, 40.45, 36.67, 26.24, 24.66, 15.93 ppm.

4-(4-(1-(4-Trifluoromethyl-benzyl)-1H-indol-3-yl)butanamido)-N-hydroxybenzamide (**I4**). HRMS (AP-ESI) *m/z* calcd for C_27_H_24_F_3_N_3_O_3_ [M + H]^+^ 496.18033, found 496.18274. ^1^H NMR (400 MHz, DMSO) *δ* 11.09 (s, 1H), 10.11 (s, 1H), 8.93 (s, 1H), 7.71–7.64 (m, 6H), 7.57 (d, *J* = 7.8 Hz, 1H), 7.38–7.33 (m, 4H), 7.11–7.07 (m, 1H), 7.03–7.00 (m, 1H), 5.48 (s, 2H), 2.76 (t, *J* = 7.2 Hz, 2H), 2.42 (t, *J* = 7.2 Hz, 2H), 2.01–1.98 (m, 2H); ^13^C NMR (400 MHz, DMSO): *δ* = 172.03, 164.40, 143.83, 142.34, 136.55, 128.48, 128.30, 128.12, 128.00, 127.39, 126.76, 125.91, 121.85, 119.31, 119.16, 118.73, 114.88, 110.41, 48.81, 36.64, 26.14, 24.62 ppm.

4-(4-(1-(2-Trifluoromethyl-benzyl)-1H-indol-3-yl)butanamido)-N-hydroxybenzamide (**I5**). HRMS (AP-ESI) *m/z* calcd for C_27_H_24_F_3_N_3_O_3_ [M + H]^+^ 496.18033, found 496.18295. ^1^H NMR (400 MHz, DMSO) *δ* 11.08 (s, 1H), 10.11 (s, 1H), 8.93 (s, 1H), 7.80 (d, *J* = 6.9 Hz, 1H), 7.71–7.61 (m, 5H), 7.52–7.45 (m, 2H), 7.29 (s, 1H), 7.18 (d, *J* = 8.0 Hz, 1H), 7.12–7.03 (m, 2H), 6.52 (d, *J* = 7.0 Hz, 1H), 5.58 (s, 2H), 2.79 (t, *J* = 7.6 Hz, 2H), 2.43 (t, *J* = 7.2 Hz, 2H), 2.04–1.97 (m, 2H); ^13^C NMR (400 MHz, DMSO): *δ* = 172.04, 164.41, 142.35, 137.40, 136.65, 133.48, 128.35, 128.26, 128.13, 127.80, 127.39, 127.12, 126.36, 123.58, 122.12, 119.46, 119.36, 118.74, 115.12, 110.04, 45.95, 36.63, 26.16, 24.61 ppm.

4-(4-(1-(3,4-Difluoro-benzyl)-1H-indol-3-yl)butanamido)-N-hydroxybenzamide (**I6**). HRMS (AP-ESI) *m/z* calcd for C_26_H_23_F_2_N_3_O_3_ [M + H]^+^ 464.17410, found 464.17673. ^1^H NMR (400 MHz, DMSO) *δ* 11.09 (s, 1H), 10.10 (s, 1H), 8.93 (s, 1H), 7.67 (dd, *J* = 19.2, 8.7 Hz, 4H), 7.55 (d, *J* = 7.8 Hz, 1H), 7.42 (d, *J* = 8.2 Hz, 1H), 7.39–7.27 (m, 3H), 7.12–7.08 (m, 1H), 7.03–6.99 (m, 2H), 5.35 (s, 2H), 2.75 (t, *J* = 7.4 Hz, 2H), 2.41 (t, *J* = 7.2 Hz, 2H), 2.02–1.95 (m, 2H); ^13^C NMR (400 MHz, DMSO): *δ* = 172.08, 164.46, 150.24, 148.58, 142.33, 136.69, 136.44, 128.31, 128.12, 127.40, 126.57, 121.85, 119.28, 119.16, 118.78, 117.97, 116.53, 114.89, 110.42, 48.27, 36.67, 26.19, 24.61 ppm.

4-(4-(1-(3-Fluoro-benzyl)-1H-indol-3-yl)butanamido)-N-hydroxybenzamide (**I7**). HRMS (AP-ESI) *m/z* calcd for C_26_H_24_FN_3_O_3_ [M + H]^+^ 446.18353, found 446.18613. ^1^H NMR (400 MHz, DMSO) *δ* 11.08 (s, 1H), 10.11 (s, 1H), 8.93 (s, 1H), 7.70–7.64 (m, 4H), 7.55 (d, *J* = 7.8 Hz, 1H), 7.40 (d, *J* = 8.2 Hz, 1H), 7.36–7.31 (m, 2H), 7.11–6.99 (m, 5H), 5.38 (s, 2H), 2.75 (t, *J* = 7.5 Hz, 2H), 2.41 (t, *J* = 7.2 Hz, 2H), 2.02–1.97 (m, 2H); ^13^C NMR (400 MHz, DMSO): *δ* = 172.05, 163.87, 161.45, 143.25, 141.94, 136.53, 130.97, 128.27, 128.19, 127.39, 126.72, 123.45, 119.26, 119.10, 118.74, 114.42, 114.35, 110.45, 48.77, 36.67, 26.20, 24.62 ppm.

4-(4-(1-(3-Bromo-benzyl)-1H-indol-3-yl)butanamido)-N-hydroxybenzamide (**I8**). HRMS (AP-ESI) *m/z* calcd for C_26_H_24_BrN_3_O_3_ [M + H]^+^ 506.39106, found 506.10611. ^1^H NMR (400 MHz, DMSO) *δ* 11.09 (s, 1H), 10.11 (s, 1H), 8.93 (s, 1H), 7.67 (dd, *J* = 18.4, 8.8 Hz, 4H), 7.56 (d, *J* = 7.8 Hz, 1H), 7.44–7.41 (m, 3H), 7.33 (s, 1H), 7.28–7.24 (m, 1H), 7.16 (d, *J* = 7.6 Hz, 1H), 7.12–7.08 (m, 1H), 7.03–6.99 (m, 1H), 5.37 (s, 2H), 2.75 (t, *J* = 7.5 Hz, 2H), 2.42 (t, *J* = 7.2 Hz, 2H), 2.02–1.95 (m, 2H); ^13^C NMR (400 MHz, DMSO): *δ* = 172.04, 164.44, 142.35, 141.78, 136.52, 131.20, 130.61, 130.15, 128.28, 128.13, 127.41, 126.68, 126.50, 122.24, 121.83, 119.28, 119.13, 118.76, 114.83, 110.43, 48.62, 36.68, 26.21, 24.63 ppm.

4-(4-(1-(3-Chloro-benzyl)-1H-indol-3-yl)butanamido)-N-hydroxybenzamide (**I9**). HRMS (AP-ESI) *m/z* calcd for C_26_H_24_ClN_3_O_3_ [M + H]^+^ 463.14767, found 462.15634. ^1^H NMR (400 MHz, DMSO) *δ* 11.08 (s, 1H), 10.11 (s, 1H), 8.92 (s, 1H), 7.70–7.64 (m, 4H), 7.56 (d, *J* = 7.8 Hz, 1H), 7.41 (d, *J* = 8.2 Hz, 1H), 7.35–7.29 (m, 3H), 7.25 (s, 1H), 7.13–7.08 (m, 2H), 7.03–6.99 (m, 1H), 5.38 (s, 2H), 2.75 (t, *J* = 7.2 Hz, 2H), 2.41 (t, *J* = 7.3 Hz, 2H), 2.02–1.94 (m, 2H); ^13^C NMR (400 MHz, DMSO): *δ* = 172.04, 164.41, 142.35, 141.55, 136.51, 133.59, 130.93, 128.27, 128.12, 127.71, 127.39, 127.26, 126.70, 126.12, 121.82, 119.28, 119.12, 118.73, 114.81, 110.44, 48.66, 36.67, 26.20, 24.62 ppm.

4-(4-(1-(2,6-Difluoro-benzyl)-1H-indol-3-yl)butanamido)-N-hydroxybenzamide (**I10**). HRMS (AP-ESI) *m/z* calcd for C_26_H_23_FN_3_O_3_ [M + H]^+^464.17410, found 464.17679. ^1^H NMR (400 MHz, DMSO) *δ* 11.09 (s, 1H), 10.11 (s, 1H), 8.93 (s, 1H), 7.67 (dd, *J* = 19.6, 8.4 Hz, 4H), 7.53 (d, *J* = 7.8 Hz, 1H), 7.47–7.39 (m, 2H), 7.16–7.12 (m, 4H), 7.03–6.99 (m, 1H), 5.39 (s, 2H), 2.71 (t, *J* = 7.4 Hz, 2H), 2.39 (t, *J* = 7.4 Hz, 2H), 1.98–1.91 (m, 2H); ^13^C NMR (400 MHz, DMSO): *δ* = 172.06, 164.47, 162.57, 160.10, 142.34, 136.35, 131.30, 128.12, 128.03, 127.38, 126.21, 121.91, 119.26, 119.16, 118.78, 114.91, 113.68, 112.52, 112.27, 109.79, 37.28, 36.62, 26.17, 24.52 ppm.

4-(4-(1-(3,5-Bis-trifluoromethyl-benzyl)-1H-indol-3-yl)butanamido)-N-hydroxybenzamide (**I11**). HRMS (AP-ESI) *m/z* calcd for C_28_H_23_F_6_N_3_O_3_ [M + H]^+^ 564.16772, found 564.17004. ^1^H NMR (400 MHz, DMSO) *δ* 11.08 (s, 1H), 10.10 (s, 1H), 8.93 (s, 1H), 8.01 (s, 1H), 7.88(s, 1H), 7.67 (dd, *J* = 19.4, 8.6 Hz, 4H), 7.58 (d, *J* = 7.8 Hz, 1H), 7.48 (d, *J* = 8.2 Hz, 1H), 7.42 (s, 1H), 7.14–7.10 (m, 1H), 7.05–7.01 (m, 1H), 5.58 (s, 2H), 2.76 (t, *J* = 7.4 Hz, 2H), 2.41 (t, *J* = 7.2 Hz, 2H), 1.99–1.96 (m, 2H); ^13^C NMR (400 MHz, DMSO): *δ* = 172.02, 164.41, 142.54, 142.34, 136.50, 131.32, 130.99, 130.67, 128.32, 128.12, 127.39, 126.56, 125.03, 122.32, 122.06, 119.39, 118.73, 115.38, 110.32, 48.21, 36.62, 26.25, 24.58 ppm.

4-(4-(1-Butyl-1H-indol-3-yl)butanamido)-N-hydroxybenzamide (**I12**). HRMS (AP-ESI) *m/z* calcd for C_23_H_27_N_3_O_3_ [M + H]^+^ 394.20860, found 394.21143. ^1^H NMR (400 MHz, DMSO) *δ* 11.08 (s, 1H), 10.09 (s, 1H), 8.92 (s, 1H), 7.67 (dd, *J* = 19.6, 8.4 Hz, 4H), 7.53 (d, *J* = 7.9 Hz, 1H), 7.40 (d, *J* = 8.2 Hz, 1H), 7.16 (s, 1H), 7.12–7.08 (m, 1H), 7.00–6.97 (m, 1H), 4.10 (t, *J* = 6.9 Hz, 2H), 2.72 (t, *J* = 7.2 Hz, 2H), 2.39 (t, *J* = 7.4 Hz, 2H), 2.03–1.93 (m, 2H), 1.74–1.67 (m, 2H), 1.28–1.19 (m, 2H), 0.87 (t, *J* = 7.4 Hz, 3H); ^13^C NMR (400 MHz, DMSO): *δ* = 172.06, 164.40, 142.35, 136.49, 128.11, 127.98, 127.38, 126.25, 121.37, 119.12, 118.72, 118.61, 113.81, 110.09, 45.40, 36.66, 32.46, 26.25, 24.64, 20.03, 14.06 ppm.

4-(4-(1-Methyl-1H-indol-3-yl)butanamido)-N-hydroxybenzamide (**I13**). HRMS (AP-ESI) *m/z* calcd for C_20_H_21_N_3_O_3_ [M + H]^+^ 352.16165, found 352.16403. ^1^H NMR (400 MHz, DMSO) *δ* 11.09 (s, 1H), 10.11 (s, 1H), 8.93 (s, 1H), 7.68 (dd, *J* = 18.4, 8.8 Hz, 4H), 7.54 (d, *J* = 7.8 Hz, 1H), 7.37 (d, *J* = 8.2 Hz, 1H), 7.15–7.12 (m, 2H), 7.03–6.99 (m, 1H), 3.73 (s, 3H), 2.73 (t, *J* = 7.4 Hz, 2H), 2.40 (t, *J* = 7.3 Hz, 2H), 2.01–1.93 (m, 2H); ^13^C NMR (400 MHz, DMSO): *δ* = 172.04, 164.41, 142.34, 137.15, 128.11, 127.94, 127.37, 127.31, 121.46, 119.02, 118.72, 118.70, 113.75, 110.00, 36.61, 32.67, 26.26, 24.56 ppm.

Preparation of **I1c** and its analogues: Derivatives **I2c**, **I3c**, **I7c**, **I10c**, **I12c**, and **I13c** were prepared as described for **I1c** (see below).

4-(4-(1H-indol-3-yl)-butyrylamino)-N-benzoylhydrazine (**I1c**). Compound methyl 4-(4-(1H-indol-3-yl)butanamido)benzoate (I1b) and hydrazine hydrate were mixed in the methanol. Then, the mixture was heated up to 80 °C and held at 80 °C with stirring for 24 h. After cooling to room temperature, the desired compound **I1c** was derived by crystallisation as white powder. HRMS *m/z*: 337.16461 [M + H]^+^. ^1^H NMR (400 MHz, DMSO) *δ* 10.79 (s, 1H), 10.10 (s, 1H), 9.64 (s, 1H), 7.78 (d, *J* = 8.7 Hz, 2H), 7.66 (d, *J* = 8.7 Hz, 2H), 7.53 (d, *J* = 7.8 Hz, 1H), 7.34 (d, *J* = 8.0 Hz, 1H), 7.14 (d, *J* = 2.1 Hz, 1H), 7.08–7.04 (m, 2H), 6.99–6.95 (m, 1H), 4.44 (s, 3H), 2.75 (t, *J* = 7.4 Hz, 2H), 2.40 (t, *J* = 7.4 Hz, 2H), 2.02–1.95 (m, 2H).

4-(4-(1-Benzyl-1H-indol-3-yl)butanamido)-N-benzoylhydrazine (**I2c**). HRMS *m/z*: 427.20109 [M + H]^+^. ^1^H NMR (400 MHz, DMSO) *δ* 10.12 (s, 1H), 9.64 (s, 1H), 7.77 (d, *J* = 8.6 Hz, 2H), 7.65 (d, *J* = 8.6 Hz, 2H), 7.55 (d, *J* = 7.8 Hz, 1H), 7.39 (d, *J* = 8.2 Hz, 1H), 7.31–7.18 (m, 6H), 7.10–7.06 (m, 1H), 7.02–6.98 (m, 1H), 5.35 (s, 2H), 4.44 (s, 2H), 2.75 (t, *J* = 7.4 Hz, 2H), 2.42 (t, *J* = 7.4 Hz, 2H), 2.02–1.95 (m, 2H).

4-(4-(1-Ethyl-1H-indol-3-yl)butanamido)-N-benzoylhydrazine (**I3c**). HRMS *m/z*: 365.19595 [M + H]^+^. ^1^H NMR (400 MHz, DMSO) *δ* 10.09 (s, 1H), 9.62 (s, 1H), 7.76 (d, *J* = 8.5 Hz, 2H), 7.64 (d, *J* = 8.5 Hz, 2H), 7.53 (d, *J* = 7.8 Hz, 1H), 7.40 (d, *J* = 8.2 Hz, 1H), 7.18 (s, 1H), 7.13–7.09 (m, 1H), 7.01–6.97 (m, 1H), 4.43 (s, 2H), 4.14 (dd, *J* = 14.3, 7.1 Hz, 2H), 2.73 (t, *J* = 7.3 Hz, 2H), 2.40 (t, *J* = 7.3 Hz, 2H), 2.00–1.93 (m, 2H), 1.33 (t, *J* = 7.2 Hz, 3H).

4-(4-(1-(3-Fluoro-benzyl)-1H-indol-3-yl)butanamido)-N-benzoylhydrazine (**I7c**). HRMS *m/z*: 445.20258 [M + H]^+^. ^1^H NMR (400 MHz, DMSO) *δ* 10.10 (s, 1H), 9.62 (s, 1H), 7.76 (d, *J* = 8.5 Hz, 2H), 7.65 (d, *J* = 8.6 Hz, 2H), 7.56 (d, *J* = 7.8 Hz, 1H), 7.39 (d, *J* = 8.2 Hz, 1H), 7.36–7.31 (m, 2H), 7.11–7.00 (m, 5H), 5.38 (s, 2H), 4.42 (s, 2H), 2.75 (t, *J* = 7.2 Hz, 2H), 2.41 (t, *J* = 7.2 Hz, 2H), 2.00–1.97 (m, 2H).

4-(4-(1-(2,6-Difluoro-benzyl)-1H-indol-3-yl)butanamido)-N-benzoylhydrazine (**I10c**). HRMS *m/z*: 436.19232 [M + H]^+^. ^1^H NMR (400 MHz, DMSO) *δ* 10.09 (s, 1H), 9.63 (s, 1H), 7.75 (d, *J* = 8.6 Hz, 2H), 7.64 (d, *J* = 8.6 Hz, 2H), 7.53 (d, *J* = 7.9 Hz, 1H), 7.47–7.40 (m, 2H), 7.16–7.12 (m, 4H), 7.03–7.00 (m, 1H), 5.39 (s, 2H), 4.43 (s, 2H), 2.71 (t, *J* = 7.4 Hz, 2H), 2.39 (t, *J* = 7.3 Hz, 2H), 1.98–1.90 (m, 2H).

4-(4-(1-Butyl-1H-indol-3-yl)butanamido)-N-benzoylhydrazine (**I12c**). HRMS *m/z*: 393.22568 [M + H]^+^. ^1^H NMR (400 MHz, DMSO) *δ* 10.10 (s, 1H), 9.63 (s, 1H), 7.76 (d, *J* = 8.5 Hz, 2H), 7.64 (d, *J* = 8.5 Hz, 2H), 7.53 (d, *J* = 7.6 Hz, 1H), 7.40 (d, *J* = 8.2 Hz, 1H), 7.16 (s, 1H), 7.13–7.09 (m, 1H), 7.01–6.97 (m, 1H), 4.10 (t, *J* = 6.8 Hz, 2H), 2.73 (t, *J* = 7.2 Hz, 2H), 2.40 (t, *J* = 7.4 Hz, 2H`), 2.00–1.93 (m, 2H), 1.74–1.67 (m, 2H), 1.29–1.19 (m, 2H), 0.87 (d, *J* = 7.4 Hz, 2H).

4-(4-(1-methyl-1H-indol-3-yl)butanamido)-N-benzoylhydrazine (**I13c**). HRMS *m/z*: 351.18027 [M + H]^+^. ^1^H NMR (400 MHz, DMSO) *δ* 10.08 (s, 1H), 9.63 (s, 1H), 7.76 (d, *J* = 8.7 Hz, 2H), 7.64 (d, *J* = 8.7 Hz, 2H), 7.53 (d, *J* = 7.8 Hz, 1H), 7.37 (d, *J* = 8.2 Hz, 1H), 7.15–7.11 (m, 2H), 7.02–6.98 (m, 1H), 4.49 (s, 2H), 3.73 (s, 3H), 2.73 (t, *J* = 7.4 Hz, 2H), 2.39 (t, *J* = 7.4 Hz, 2H`), 2.00–1.92 (m, 2H).

Preparation of **I1e1** and its analogues: derivatives **I1e2–I1e4**, **I2e2**, **I2e3**, **I3e2**, **I3e3**, **I7e2**, **I10e2**, **I12e2**, **I12e3**, **I13e2**, and **I13e3** were prepared as described for **I1e1** (see below).

N-[4-(N′-Ethyl-hydrazinocarbonyl)-phenyl]-4-(1H-indol-3-yl)-butyramide (**I1e1**). HRMS (AP-ESI) *m/z* calcd for C_21_H_24_N_4_O_2_ [M + H]^+^ 365.19328, found 365.19626. ^1^H NMR (400 MHz, DMSO) *δ* 10.79 (s, 1H), 10.12 (s, 1H), 9.89 (d, *J* = 6.3 Hz, 1H), 7.77 (d, *J* = 8.2 Hz, 2H), 7.66 (d, *J* = 8.2 Hz, 2H), 7.52 (d, *J* = 7.7 Hz, 1H), 7.33 (d, *J* = 8.0 Hz, 1H), 7.13 (s, 1H), 7.08–7.04 (m, 1H), 6.98–6.95 (m, 1H), 5.02 (d, *J* = 6.0 Hz, 1H), 2.81–2.72 (m, 4H), 2.40 (t, *J* = 7.0 Hz, 2H), 1.99–1.95 (m, 2H), 1.03 (t, *J* = 7.0 Hz, 3H); ^13^C NMR (400 MHz, DMSO): *δ* = 172.14, 165.38, 142.48, 136.78, 128.29, 127.77, 127.62, 122.80, 121.29, 118.78, 118.63, 118.58, 114.40, 111.81, 46.02, 36.67, 26.25, 24.76, 13.59 ppm.

4-(1H-Indol-3-yl)-N-[4-(N′-propyl-hydrazinocarbonyl)-phenyl]-butyramide (**I1e2**). HRMS (AP-ESI) *m/z* calcd for C_22_H_26_N_4_O_2_ [M + H]^+^ 379.20893, found 379.21191. ^1^H NMR (400 MHz, DMSO) *δ* 10.78 (s, 1H), 10.09 (s, 1H), 9.88 (d, *J* = 6.5 Hz, 1H), 7.76 (d, *J* = 8.6 Hz, 2H), 7.65 (d, *J* = 8.6 Hz, 2H), 7.52 (d, *J* = 7.7 Hz, 1H), 7.33 (d, *J* = 8.0 Hz, 1H), 7.13 (s, 1H), 7.07–7.04 (m, 1H), 6.98–6.94 (m, 1H), 5.06–5.01 (m, 1H), 2.75–2.72 (m, 4H), 2.39 (t, *J* = 7.2 Hz, 2H), 2.01–1.94 (m, 2H), 1.50–1.41 (m, 2H), 0.90 (t, *J* = 7.4 Hz, 3H); ^13^C NMR (400 MHz, DMSO): *δ* = 172.11, 165.37, 142.44, 136.79, 128.31, 127.83, 127.64, 122.81, 121.31, 118.79, 118.65, 118.60, 114.41, 111.81, 53.64, 36.67, 26.23, 24.75, 21.34, 12.15 ppm.

N-[4-(N′-Butyl-hydrazinocarbonyl)-phenyl]-4-(1H-indol-3-yl)-butyramide (**I1e3**). HRMS (AP-ESI) *m/z* calcd for C_23_H_28_N_4_O_2_ [M + H]^+^ 393.22458, found 393.22726. ^1^H NMR (400 MHz, DMSO) *δ* 10.78 (s, 1H), 10.10 (s, 1H), 9.88 (d, *J* = 6.6 Hz, 1H), 7.76 (d, *J* = 8.6 Hz, 2H), 7.65 (d, *J* = 8.6 Hz, 2H), 7.52 (d, *J* = 7.8 Hz, 1H), 7.33 (d, *J* = 8.0 Hz, 1H), 7.13 (s, 1H), 7.07–7.04 (m, 1H), 6.98–6.94 (m, 1H), 5.03–4.99 (m, 1H), 2.78–2.72 (m, 4H), 2.39 (t, *J* = 7.3 Hz, 2H), 2.01–1.93 (m, 2H), 1.46–1.30 (m, 4H), 0.89 (t, *J* = 7.2 Hz, 3H); ^13^C NMR (400 MHz, DMSO): *δ* = 172.10, 165.35, 142.43, 136.78, 128.30, 127.81, 127.63, 122.81, 121.31, 118.79, 118.63, 118.59, 114.40, 111.81, 51.44, 36.66, 30.27, 26.23, 24.75, 20.32, 14.41 ppm.

4-(1H-Indol-3-yl)-N-[4-(N′-isobutyl-hydrazinocarbonyl)-phenyl]-butyramide (**I1e4**). HRMS (AP-ESI) *m/z* calcd for C_23_H_28_N_4_O_2_ [M + H]^+^ 393.22458, found 393.22717. ^1^H NMR (400 MHz, DMSO) *δ* 10.78 (s, 1H), 10.10 (s, 1H), 9.88 (d, *J* = 6.3 Hz, 1H), 7.76 (d, *J* = 8.6 Hz, 2H), 7.65 (d, *J* = 8.6 Hz, 2H), 7.52 (d, *J* = 7.8 Hz, 1H), 7.33 (d, *J* = 8.1 Hz, 1H), 7.13 (s, 1H), 7.07–7.04 (m, 1H), 6.98–6.94 (m, 1H), 5.06–5.01 (m, 1H), 2.73 (t, *J* = 7.4 Hz, 2H), 2.59 (t, *J* = 6.2 Hz, 2H), 2.39 (t, *J* = 7.4 Hz, 2H), 2.01–1.93 (m, 2H), 1.79–1.69 (m, 1H), 0.92 (d, *J* = 6.6 Hz, 6H); ^13^C NMR (400 MHz, DMSO): *δ* = 172.10, 165.34, 142.43, 136.78, 128.31, 127.82, 127.63, 122.81, 121.31, 118.79, 118.63, 118.59, 114.40, 111.81, 59.75, 36.67, 26.99, 26.23, 24.75, 21.13, 20.17 ppm.

4-(1-Benzyl-1H-indol-3-yl)-N-[4-(N′-propyl-hydrazinocarbonyl)-phenyl]-butyramide (**I2e2**). HRMS (AP-ESI) *m/z* calcd for C_29_H_32_N_4_O_2_ [M + H]^+^ 469.25588, found 469.25735. ^1^H NMR (400 MHz, DMSO) *δ* 10.10 (s, 1H), 9.88 (s, 1H), 7.77 (d, *J* = 8.8 Hz, 2H), 7.65 (d, *J* = 8.8 Hz, 2H), 7.55 (d, *J* = 7.8 Hz, 1H), 7.39 (d, *J* = 8.2 Hz, 1H), 7.31–7.27 (m, 3H), 7.23 (d, *J* = 7.2 Hz, 1H), 7.20–7.18 (m, 2H), 7.10–7.06 (m, 1H), 7.01–6.98 (m, 1H), 5.35 (s, 2H), 5.04 (s, 1H), 2.77–2.73 (m, 4H), 2.41 (t, *J* = 7.4 Hz, 2H), 2.02–1.95 (m, 2H), 1.50–1.41 (m, 2H), 0.91 (t, *J* = 7.4 Hz, 3H); ^13^C NMR (400 MHz, DMSO): *δ* = 172.05, 165.35, 142.42, 138.91, 136.59, 128.96, 128.30, 128.26, 127.85, 127.70, 127.46, 126.72, 121.64, 119.19, 118.94, 118.66, 114.51, 110.49, 53.64, 49.37, 36.68, 26.21, 24.65, 21.34, 12.15 ppm.

4-(1-Benzyl-1H-indol-3-yl)-N-[4-(N′-butyl-hydrazinocarbonyl)-phenyl]-butyramide (**I2e3**). HRMS (AP-ESI) *m/z* calcd for C_30_H_34_N_4_O_2_ [M + H]^+^ 483.27153, found 483.27390. ^1^H NMR (400 MHz, DMSO) *δ* 10.10 (s, 1H), 9.88 (d, *J* = 6.5 Hz, 1H), 7.76 (d, *J* = 8.8 Hz, 2H), 7.65 (d, *J* = 8.8 Hz, 2H), 7.55 (d, *J* = 7.8 Hz, 1H), 7.39 (d, *J* = 8.2 Hz, 1H), 7.31–7.27 (m, 3H), 7.23 (d, *J* = 7.2 Hz, 1H), 7.20–7.18 (m, 2H), 7.10–7.06 (m, 1H), 7.01–6.98 (m, 1H), 5.35 (s, 2H), 5.00 (dd, *J* = 12.4, 6.0 Hz, 1H), 2.78–2.73 (m, 4H), 2.41 (t, *J* = 7.4 Hz, 2H), 2.02–1.94 (m, 2H), 1.50–1.30 (m, 4H), 0.89 (t, *J* = 7.4 Hz, 3H); ^13^C NMR (400 MHz, DMSO): *δ* = 172.04, 165.34, 142.41, 138.92, 136.58, 128.96, 128.29, 128.26, 127.84, 127.71, 127.46, 126.72, 121.64, 119.19, 118.94, 118.65, 114.50, 110.49, 51.44, 49.37, 36.68, 30.27, 26.21, 24.64, 20.31, 14.40 ppm.

4-(1-Ethyl-1H-indol-3-yl)-N-[4-(N′-propyl-hydrazinocarbonyl)-phenyl]-butyramide (**I3e2**). HRMS (AP-ESI) *m/z* calcd for C_24_H_30_N_4_O_2_ [M + H]^+^ 407.24023, found 407.24222. ^1^H NMR (400 MHz, DMSO) *δ* 10.09 (s, 1H), 9.88 (d, *J* = 5.2 Hz, 1H), 7.76 (d, *J* = 8.8 Hz, 2H), 7.65 (d, *J* = 8.8 Hz, 2H), 7.53 (d, *J* = 7.8 Hz, 1H), 7.40 (d, *J* = 8.2 Hz, 1H), 7.18 (s, 1H), 7.13–7.09 (m, 1H), 7.01–6.97 (m, 1H), 5.04 (d, *J* = 5.3 Hz, 1H), 2.73 (t, *J* = 7.4 Hz, 4H), 2.40 (t, *J* = 7.2 Hz, 2H), 2.01–1.95 (m, 2H), 1.50–1.41 (m, 2H), 1.33 (t, *J* = 7.2 Hz, 3H), 0.91 (t, *J* = 7.6 Hz, 3H); ^13^C NMR (400 MHz, DMSO): *δ* = 172.06, 165.34, 142.41, 136.16, 128.30, 128.07, 127.82, 125.58, 121.38, 119.14, 118.66, 118.62, 113.96, 109.99, 53.62, 40.45, 36.67, 26.23, 24.67, 21.33, 15.93, 12.15 ppm.

N-[4-(N′-Butyl-hydrazinocarbonyl)-phenyl]-4-(1-ethyl-1H-indol-3-yl)-butyramide (**I3e3**). HRMS (AP-ESI) *m/z* calcd for C_25_H_32_N_4_O_2_ [M + H]^+^ 421.25588, found 421.25818. ^1^H NMR (400 MHz, DMSO) *δ* 10.09 (s, 1H), 9.88 (d, *J* = 6.6 Hz, 1H), 7.76 (d, *J* = 8.8 Hz, 2H), 7.65 (d, *J* = 8.8 Hz, 2H), 7.53 (d, *J* = 7.8 Hz, 1H), 7.40 (d, *J* = 8.2 Hz, 1H), 7.18 (s, 1H), 7.13–7.09 (m, 1H), 7.01–6.97 (m, 1H), 5.01 (dd, *J* = 12.4, 6.0 Hz, 1H), 4.14 (q, *J* = 7.2 Hz, 2H), 2.79–2.71 (m, 4H), 2.40 (t, *J* = 7.4 Hz, 2H), 2.01–1.93 (m, 2H), 1.47–1.35 (m, 4H), 1.33 (t, *J* = 7.2 Hz, 3H), 0.89 (t, *J* = 7.2 Hz, 3H); ^13^C NMR (400 MHz, DMSO): *δ* = 172.06, 165.33, 142.42, 136.16, 128.30, 128.07, 127.81, 125.58, 121.38, 119.14, 118.65, 118.63, 113.96, 109.99, 51.43, 40.45, 36.67, 30.27, 26.23, 24.67, 20.31, 15.93, 14.41 ppm.

4-[1-(3-Fluoro-benzyl)-1H-indol-3-yl]-N-[4-(N′-propyl-hydrazinocarbonyl)-phenyl]-butyramide (**I7e2**). HRMS (AP-ESI) *m/z* calcd for C_29_H_31_FN_4_O_2_ [M + H]^+^ 487.24646, found 487.24854. ^1^H NMR (400 MHz, DMSO) *δ* 10.11 (s, 1H), 9.88 (d, *J* = 6.6 Hz, 1H), 7.76 (d, *J* = 8.8 Hz, 2H), 7.65 (d, *J* = 8.8 Hz, 2H), 7.56 (d, *J* = 7.7 Hz, 1H), 7.40 (d, *J* = 8.2 Hz, 1H), 7.34–7.31 (m, 2H), 7.11–6.99 (m, 5H), 5.38 (s, 2H), 5.04 (dd, *J* = 12.4, 6.0 Hz, 1H), 2.77–2.70 (m, 4H), 2.42 (t, *J* = 7.4 Hz, 2H), 2.02–1.95 (m, 2H), 1.50–1.41 (m, 2H), 0.91 (t, *J* = 7.4 Hz, 3H); ^13^C NMR (400 MHz, DMSO): *δ* = 172.05, 165.36, 161.45, 142.39, 141.87, 136.54, 131.05, 130.96, 128.29, 127.85, 126.71, 123.47, 121.78, 119.26, 119.09, 118.66, 114.77, 114.41, 114.09, 110.44, 53.64, 48.79, 36.68, 26.20, 24.63, 21.33, 12.14 ppm.

4-[1-(2,6-Difluoro-benzyl)-1H-indol-3-yl]-N-[4-(N′-propyl-hydrazinocarbonyl)-phenyl]-butyramide (**I10e2**). HRMS (AP-ESI) *m/z* calcd for C_29_H_30_F_2_N_4_O_2_ [M + H]^+^ 505.23704, found 505.23914. ^1^H NMR (400 MHz, DMSO) *δ* 10.08 (s, 1H), 9.88 (d, *J* = 5.4 Hz, 1H), 7.76 (d, *J* = 8.8 Hz, 2H), 7.64 (d, *J* = 8.7 Hz, 2H), 7.53 (d, *J* = 7.8 Hz, 1H), 7.43–7.39 (m, 2H), 7.16–7.12 (m, 4H), 7.03–6.99 (m, 1H), 5.39 (s, 2H), 5.04 (d, *J* = 5.3 Hz, 1H), 2.75–2.69 (m, 4H), 2.39 (t, *J* = 7.2 Hz, 2H), 1.98–1.90 (m, 2H), 1.50–1.41 (m, 2H), 0.91 (t, *J* = 7.4 Hz, 3H); ^13^C NMR (400 MHz, DMSO): *δ* = 172.00, 165.34, 160.03, 142.39, 136.36, 131.30, 128.29, 128.04, 127.85, 126.22, 121.90, 119.26, 119.15, 118.65, 114.90, 113.69, 112.53, 112.28, 109.79, 53.63, 37.29, 36.63, 26.14, 24.53, 21.33, 12.14 ppm.

4-(1-Butyl-1H-indol-3-yl)-N-[4-(N′-propyl-hydrazinocarbonyl)-phenyl]-butyramide (**I12e2**). HRMS (AP-ESI) *m/z* calcd for C_26_H_34_N_4_O_2_ [M + H]^+^ 435.27153, found 435.27414. ^1^H NMR (400 MHz, DMSO) *δ* 10.09 (s, 1H), 9.88 (d, *J* = 5.3 Hz, 1H), 7.76 (d, *J* = 8.8 Hz, 2H), 7.65 (d, *J* = 8.8 Hz, 2H), 7.53 (d, *J* = 7.8 Hz, 1H), 7.40 (d, *J* = 8.2 Hz, 1H), 7.16 (s, 1H), 7.12–7.08 (m, 1H), 7.00–6.97 (m, 1H), 5.04 (d, *J* = 5.2 Hz, 1H), 4.10 (t, *J* = 7.0 Hz, 2H), 2.75–2.71 (m, 4H), 2.40 (t, *J* = 7.4 Hz, 2H), 2.00–1.93 (m, 2H), 1.74–1.67 (m, 2H), 1.50–1.41 (m, 2H), 1.29–1.19 (m, 2H), 0.91 (t, *J* = 7.4 Hz, 3H), 0.88 (t, *J* = 7.4 Hz, 3H); ^13^C NMR (400 MHz, DMSO): *δ* = 172.11, 165.36, 142.40, 136.50, 128.29, 127.99, 127.38, 126.24, 121.38, 119.11, 118.67, 118.61, 113.82, 110.08, 53.63, 45.40, 36.67, 32.45, 26.24, 24.63, 21.31, 20.03, 14.04, 12.13 ppm.

N-[4-(N′-Butyl-hydrazinocarbonyl)-phenyl]-4-(1-butyl-1H-indol-3-yl)-butyramide (**I12e3**). HRMS (AP-ESI) *m/z* calcd for C_27_H_36_N_4_O_2_ [M + H]^+^ 449.28718, found 449.28943. ^1^H NMR (400 MHz, DMSO) *δ* 10.09 (s, 1H), 9.88 (d, *J* = 6.6 Hz, 1H), 7.76 (d, *J* = 8.7 Hz, 2H), 7.65 (d, *J* = 8.8 Hz, 2H), 7.53 (d, *J* = 7.8 Hz, 1H), 7.40 (d, *J* = 8.2 Hz, 1H), 7.16 (s, 1H), 7.12–7.09 (m, 1H), 7.00–6.97 (m, 1H), 5.01 (dd, *J* = 12.4, 6.0 Hz, 1H), 4.10 (t, *J* = 6.8 Hz, 2H), 2.78–2.71 (m, 4H), 2.39 (t, *J* = 7.4 Hz, 2H), 2.00–1.93 (m, 2H), 1.74–1.67 (m, 2H), 1.47–1.19 (m, 6H), 0.90–0.86 (m, 6H); ^13^C NMR (400 MHz, DMSO): *δ* = 172.11, 165.36, 142.41, 136.50, 128.28, 127.99, 127.82, 126.23, 121.37, 119.11, 118.68, 118.61, 113.83, 110.07, 51.44, 45.40, 36.67, 32.45, 30.25, 26.25, 24.63, 20.03, 20.01, 14.38, 14.04 ppm.

4-(1-Methyl-1H-indol-3-yl)-N-[4-(N′-propyl-hydrazinocarbonyl)-phenyl]-butyramide (**I13e2**). HRMS (AP-ESI) *m/z* calcd for C_23_H_28_N_4_O_2_ [M + H]^+^ 393.22458, found 393.22690. ^1^H NMR (400 MHz, DMSO) *δ* 10.08 (s, 1H), 9.87 (d, *J* = 6.3 Hz, 1H), 7.76 (d, *J* = 8.8 Hz, 2H), 7.65 (d, *J* = 8.8 Hz, 2H), 7.53 (d, *J* = 7.8 Hz, 1H), 7.37 (d, *J* = 8.2 Hz, 1H), 7.15–7.11 (m, 1H), 7.02–6.98 (m, 1H), 5.03 (dd, *J* = 11.6, 5.6 Hz, 1H), 3.73 (s, 3H), 2.75–2.71 (m, 4H), 2.39 (t, *J* = 7.4 Hz, 2H), 2.00–1.93 (m, 2H), 1.50–1.41 (m, 2H), 0.90 (t, *J* = 7.4 Hz, 3H); ^13^C NMR (400 MHz, DMSO): *δ* = 172.05, 165.35, 142.42, 137.16, 128.30, 127.95, 127.83, 127.29, 121.45, 119.02, 118.69, 118.63, 113.77, 109.98, 53.63, 36.62, 32.66, 26.25, 24.57, 21.34, 12.15 ppm.

N-[4-(N′-Butyl-hydrazinocarbonyl)-phenyl]-4-(1-methyl-1H-indol-3-yl)-butyramide (**I13e3**). HRMS (AP-ESI) *m/z* calcd for C_23_H_28_N_4_O_2_ [M + H]^+^ 407.24023, found 407.24243. ^1^H NMR (400 MHz, DMSO) *δ* 10.09 (s, 1H), 9.88 (d, *J* = 6.4 Hz, 1H), 7.76 (d, *J* = 8.8 Hz, 2H), 7.65 (d, *J* = 8.8 Hz, 2H), 7.53 (d, *J* = 7.8 Hz, 1H), 7.37 (d, *J* = 8.2 Hz, 1H), 7.15–7.11 (m, 1H), 7.02–6.98 (m, 1H), 5.01 (q, *J* = 6.0 Hz, 1H), 3.73 (s, 3H), 2.79–2.71 (m, 4H), 2.39 (t, *J* = 7.4 Hz, 2H), 2.00–1.93 (m, 2H), 1.52–1.30 (m, 4H), 0.89 (t, *J* = 7.2 Hz, 3H); ^13^C NMR (400 MHz, DMSO): *δ* = 172.05, 165.34, 142.42, 137.16, 128.30, 127.95, 127.81, 127.30, 121.45, 119.02, 118.69, 118.63, 113.76, 109.98, 51.44, 36.62, 32.66, 30.27, 26.25, 24.57, 20.32, 14.41 ppm.

### *In vitro* HDACs inhibitory assay

All of the HDAC enzymes were purchased from BPS Bioscience (San Diego, CA). Briefly, 20 μl of HeLa nucleus extract or recombinant HDAC enzyme solution was mixed with various concentrations of tested compound (20 μl). The mixture was incubated at 30 °C for 1 h (for the time-dependent assay, the mixture was incubated for 15, 30, 60, and 90 min, respectively), then 10 μl of fluorogenic substrate (Boc-Lys (acetyl)-AMC (3 mM) for HDAC1, 2, 3, and 6, Boc-Lys (trifluoroacetyl)-AMC (3 mM) for HDAC 4, 7, 8, and 9) was added. After incubation at 30 °C for 2 h, the catalytic reaction was stopped by addition of 10 μl of developer containing trypsin and trichostatin A (TSA). After 30 min, fluorescence intensity was measured using a microplate reader at excitation and emission wavelengths of 360 and 460 nm, respectively. The inhibition ratios were calculated from the fluorescence intensity readings of tested wells relative to those of control wells, and the IC_50_ curves and values were determined by GraphPad Prism 6.0 software (La Jolla, CA).

### *In vitro* antiproliferative activity

Tumour cell inhibition was determined using the MTT assay using SAHA as the control. U266 and U937 cells were cultured in RPMI-1640 medium supplemented with 10% FBS. The stock solutions of tested compounds were diluted with culture medium. Briefly, the cells were seeded into each well of 96-well plate, which were incubated at 37 °C, 5% CO_2_ until confluency 90–95%. Then, the cells were treated with compound sample at various concentrations for 48 h. Afterward, 20 MTT working solution (5 mg/ml) was added to each well and incubated for another 4 h. After incubation, the medium formed from MTT was extracted by adding DMSO (200 µl). The optical density (OD) at 490 nm and 630 nm were measured by Universal Microplate Spectrophotometer. The percentage of cell growth inhibition was calculated according to the formula: % inhibition = [1 – (Sample group OD_490_ – Sample group OD_630_)/(Control group OD_490_ – Control group OD_630_)] × 100%. The IC_50_ values were calculated with Origin 7.5 software (Originlab Corporation, Northampton, MA), and standard deviations of the IC_50_ values were obtained through at least 3 independent experiments.

### *In vivo* antitumor activity

For *in vivo* antitumor efficacy study, 1.8 × 10^7^ HepG2 cells were inoculated subcutaneously in the right shoulder of male athymic nude mice (5–6 weeks old, Slac Laboratory Animal, Shanghai, China). Then days after injection, tumours were palpable and mice were randomised into treatment and control groups (seven mice per group). The treatment groups were administrated with 100 mg/kg/d of SAHA and 50 mg/kg/d of **I13** intragastrically, the control group was administrated with equal volume of PBS solution. During treatment, the body weight of mice was monitored regularly. After 16 days of administration, the mice were executed and the tumour weight was measured by an electronic balance.

### Annexin V/PI detection

HepG2 cells in logarithmic growth phase were seeded in six-well plates (4 × 10^5^ cells/well) and incubated with different doses of FNA and SAHA (0.2 and 0.8 µM) for 24 h. After the incubation, cells were washed with PBS, collected, resuspended with binding buffer from the Annexin V-FITC kit (Thermo Fisher Co., Waltham, MA), and then added with 5 µl annexin V-FITC and mixed gently. After 10 min of incubation, 1 µl PI was added to each sample and mixed gently. After incubation at room temperature for another 20 min in the dark, cells were subjected to flow cytometer (CytoFLEX, Beckman Coulter, Brea, CA).

### Statistics

Statistics were performed with SPSS 17.0 (SPSS Inc., Chicago, IL). Analysis of variance (ANOVA) was used to determine differences among groups. When ANOVA detected significant results, least significant difference (LSD) tests were utilised to compare between groups. Results were considered statistically significant when *p* < 0.05.

## Results and discussion

### Chemistry

**Scheme 1.**
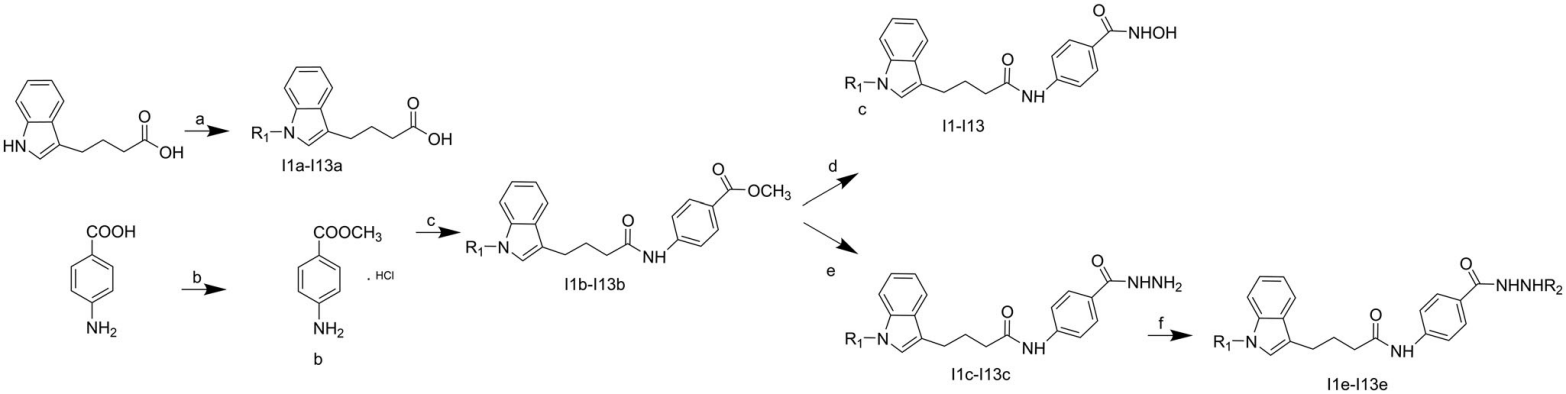
Synthesis of target compounds. Reagents and conditions: (a) substituted bromobenzene, NaH, DMF, Ar_2_, 0 °C-rt, 3 h; (b) (CO)_2_Cl, MeOH, 0 °C, overnight; (c) TBTU, Et_3_N, DCM, 0 °C-rt, overnight; (d) NH_2_OK, MeOH, 2 h; (e) NH_2_NH_2_.H_2_O, MeOH, reflux, 12–48 h; (f) aliphatic aldehydes, NaBH_4_, rt, 4 h.

Based on lead structure IBHA, a series of indol-3-ylbutyric acid derivatives were synthesised according to the procedures described in Scheme 1. The starting material IBA was coupled with a series of bromobenzene. The other material, 4-aminobenzoic acid, was protected by a methyl ester. The intermediates I1b–I13b were derived by conjugation of b and I1a–I13a. Target molecules I1–I17 were synthesised by treatment of corresponding intermediates (I1b–I13b) with NH_2_OK in methanol. The hydrazide compounds were generated by hydrazinolysis, condensation, and reduction reactions.

### Biological studies

#### HDAC inhibitory activity assay

To investigate the enzyme inhibitory potency of the synthesised molecules, an enzymatic assay was performed using HeLa nucleus extract containing a mixture of HDAC isoforms. In the screening, percentage inhibition rate (PIR) at the concentration of 1 µM was used as a measure of compound potency. The overall result revealed that molecules with hydroxamic acids as ZBGs have better inhibitory activities (Table 1) than those with hydrazide ZBGs (Table 2). Compound **I13** exhibited the highest inhibitory potency with PIR of 82.19. Small sized alkyl substituents in the R_1_ position showed optimal HDAC inhibitory activity, such as **I3** (R_1_ of ethyl group, PIR of 78.94) and **I13** (R_1_ of methyl group, 82.19). While molecule **I12** with bigger sized n-butyl group exhibited reduced activity with PIR of 62.70. Among the aromatic substituted R_1_ groups, the 1,6-2F and 4-CF3 substitution in the phenyl ring showed good inhibitory activities with PIR values of 79.10 (**I10**) and 68.50 (**I4**), respectively. In the hydrazide series, **I1e2** and **I2e2** with n-propyl substituted R_2_ group exhibited good performance with PIR of 61.34 and 57.16, respectively, compared with SAHA (PIR of 59.91). The lysate-based enzymatic test method may affect the performance of hydrazide containing molecules. The recombinant HDAC enzyme based assay will be used for further more accurate illustration of derived HDACIs.

**Table 1.** Enzyme inhibitory and antiproliferative activities of the derived hydroxamic acid containing compounds. 
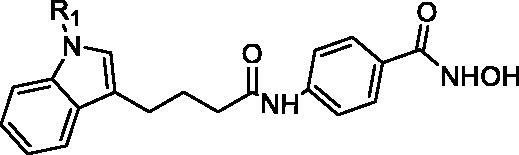

Compounds	R1	HDACs^a^	U937^b^	U266^b^	HepG2^b^
**I1**	–H	53.81 ± 1.60	1.02 ± 0.13	0.84 ± 0.09	8.98 ± 0.16
**I2**	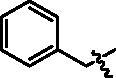	78.94 ± 3.41	0.95 ± 0.04	0.48 ± 0.09	3.63 ± 0.26
**I3**	–C_2_H_5_	78.94 ± 2.34	0.94 ± 0.04	0.24 ± 0.03	1.92 ± 0.05
**I4**	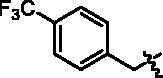	68.50 ± 1.22	0.97 ± 0.04	0.96 ± 0.08	4.45 ± 0.25
**I5**	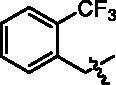	19.30 ± 1.60	4.42 ± 0.18	0.88 ± 0.07	6.17 ± 0.21
**I6**	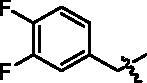	16.14 ± 1.07	1.25 ± 0.21	0.82 ± 0.06	7.72 ± 0.34
**I7**	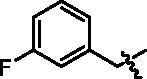	46.17 ± 2.94	0.95 ± 0.01	0.84 ± 0.06	4.87 ± 0.12
**I8**	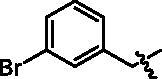	15.87 ± 3.27	1.94 ± 0.17	0.71 ± 0.06	6.45 ± 0.28
**I9**	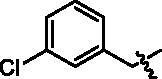	56.68 ± 2.42	1.00 ± 0.15	0.65 ± 0.04	5.67 ± 0.06
**I10**	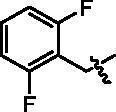	79.10 ± 4.65	0.63 ± 0.07	0.16 ± 0.07	3.39 ± 0.25
**I11**	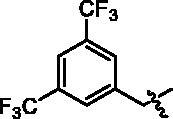	52.24 ± 1.10	4.58 ± 0.23	0.74 ± 0.05	4.76 ± 0.61
**I12**	–(CH_2_)_3_CH_3_	62.70 ± 1.63	1.01 ± 0.03	0.12 ± 0.02	5.76 ± 0.65
**I13**	–CH_3_	82.19 ± 1.60	0.93 ± 0.08	0.16 ± 0.04	1.85 ± 0.31
**SAHA**		59.91 ± 3.73	1.40 ± 0.02	0.88 ± 0.08	8.68 ± 0.29

^a^ Percentage inhibition rate (%, at the concentration of 1 µM), HeLa nucleus extract was used for the test. Each value is the mean of at least three experiments.

^b^ IC_50_, µM. Each value is the mean of at least three experiments.

**Table 2.** Enzyme inhibitory and antiproliferative activities of the derived hydrazide compounds. 
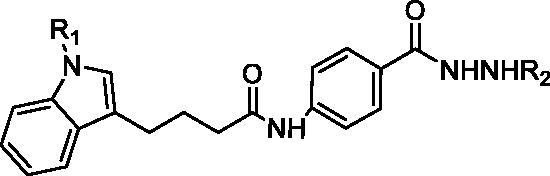

Compounds	R1	R2	HDACs^a^	U266^b^
**I1e1**	–H	–C_2_H_5_	50.60 ± 2.02	15.97 ± 0.14
**I1e2**	–H	–(CH_2_)_2_CH_3_	61.34 ± 3.56	0.64 ± 0.02
**I1e3**	–H	–(CH_2_)_3_CH_3_	30.47 ± 1.17	1.06 ± 0.05
**I1e4**	–H	–CH_2_CH (CH_3_)_2_	7.16 ± 1.50	15.98 ± 0.67
**I2e2**	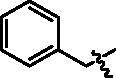	–(CH_2_)_2_CH_3_	57.46 ± 2.06	1.11 ± 0.08
**I2e3**	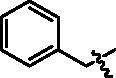	–(CH_2_)_3_CH_3_	51.41 ± 2.78	1.71 ± 0.07
**I3e2**	–C_2_H_5_	–(CH_2_)_2_CH_3_	36.51 ± 2.93	0.48 ± 0.10
**I3e3**	–C_2_H_5_	–(CH_2_)_3_CH_3_	31.94 ± 1.51	1.45 ± 0.10
**I7e2**	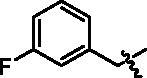	–(CH_2_)_2_CH_3_	30.45 ± 3.38	1.32 ± 0.08
**I10e2**	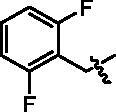	–(CH_2_)_2_CH_3_	46.85 ± 2.06	0.74 ± 0.05
**I12e2**	–(CH_2_)_3_CH_3_	–(CH_2_)_2_CH_3_	39.73 ± 1.84	1.28 ± 0.12
**I12e3**	–(CH_2_)_3_CH_3_	–(CH_2_)_3_CH_3_	42.76 ± 1.56	1.44 ± 0.31
**I13e2**	–CH_3_	–(CH_2_)_2_CH_3_	29.68 ± 2.06	0.71 ± 0.11
**I13e3**	–CH_3_	–(CH_2_)_3_CH_3_	32.15 ± 1.03	1.36 ± 0.06
**SAHA**			59.91 ± 3.73	0.88 ± 0.08

^a^ Percentage inhibition rate (%, at the concentration of 1 µM), HeLa nucleus extract was used for the test. Each value is the mean of at least three experiments.

^b^ IC_50_, µM. Each value is the mean of at least three experiments.

To evaluate the isoform selectivity of the most active molecule **I13**, HDAC1, 2, 3, 4, 6, 7, 8, and 9 were selected for the screening (Table 3). The result revealed that **I13** could effectively inhibit the activity of HDAC1, HDAC3, and HDAC6 with IC_50_ values of 13.9, 12.1, and 7.71 nM, respectively. Compared with SAHA, and the class I HDAC inhibition selective MS275, **I13** did not show inhibitory selectivity of a specific HDAC isoform or a particular class. Considering the high potency of **I13** against HDAC6, selective HDAC6 inhibitor might be derived by further structural modification of **I13**.

**Table 3.** Enzyme inhibitory activity of I13 compared with MS275 and SAHA (IC_50_, nM^a^).

HDACIs	HDAC1	HDAC2	HDAC3	HDAC4	HDAC6	HDAC7	HDAC8	HDAC9
I13	13.9	49.9	12.1	>5000	7.71	326.3	284.3	>5000
MS275	53.9	108.2	77.2	>5000	>5000	>5000	>5000	>5000
SAHA	50.7	90.4	164.1	>5000	169.5	>5000	4008	>5000

^a^ Each value is the mean of at least three experiments.

#### Antiproliferative activity

To evaluate the antiproliferative activity of hydroxamic acid containing molecules, MTT assays were performed using U937, U266, and HepG2 cell lines. Several synthesised compounds revealed improved inhibitory potency than the positive control SAHA (Table 1). Among them, molecule **I3** and **I13** which have good performance in the enzyme inhibitory assay, also showed high *in vitro* antiproliferative activity in the cancer cell based test. The IC_50_ values of compound **I3** and **I13** against U937, U266, and HepG2 cells were 0.94 and 0.93 μM, 0.24 and 0.16 μM, 1.92 and 1.85 μM, respectively, compared with SAHA (1.40, 0.88, and 8.68 μM). Molecule **I13** exhibited 5.5-fold and 4.7-fold more active than SAHA in the inhibition of U266 and HepG2 cells. Compound **I12** also showed good antiproliferative potency in the cell based screening, especially in the inhibition of U266 cells (with IC_50_ value of 0.12 μM).

The hydrazide molecules showed reduced activity in the enzyme inhibitory assay. Therefore, only the most sensitive cell line U266 was used for the test of hydrazide derivatives. As shown in Table 2, the hydrazide compounds did not exhibit improved antiproliferative activity than the corresponding molecules with hydroxamic acid ZBG. Among the hydrazide series, molecules with -propyl substituted R_2_ showed good inhibitory potency. The IC_50_ values of compound **I1e2**, **I3e2**, **I10e2**, and **I13e2** were 0.64, 0.48, 0.74, and 0.71 μM, respectively, compared with SAHA (0.88 μM).

A series of tumour cell lines including the lung cancer A549, Calu-3, SPC-A-1, H322, H1299, breast cancer MDA-MB-231, MCF-7, MDA-MB-468, colon carcinoma LoVo, Colo205, ovarian cancer A2780, SKOV3, gastric cancer MKN45, pancreatic cancer PNAC-1, leukemic K562, and multiple myeloma OPM2 cells were cultured for the antiproliferative assay of active compound **I13**. In the present study, molecule **I13** could inhibit the growth of both solid and haematologic tumour cell lines (Table 4). Against most of the tested cell lines, **I13** exhibited improved inhibitory activity compared with SAHA. In the antiproliferative test against A549, H1299, MCF-7, LoVo, Colo205, and MKN45 cells, **I13** exhibited less potency than SAHA. Remarkably, in inhibition the growth of A2780 and PNAC-1 cells, molecule **I13** was revealed to be 5.9-fold (IC_50_ values of 4.6 μM), and 5.6-fold (IC_50_ values of 1.17 μM) more active than SAHA with IC_50_ values of 27.3 μM and 6.57 μM, respectively. The antiproliferative activity of molecule **I13** was considered to be restricted by the poor enzyme inhibitory selectivity. Enhancement of selectivity could be a useful strategy to improve the anticancer activity of this group of HDACIs.

**Table 4.** Antiproliferative activity of I13 compared with SAHA (IC_50_, μM^a^).

Cell line	Tumour type	I13	SAHA
A549	Lung cancer	3.15	1.73
Calu-3	Lung cancer	1.26	3.18
SPC-A-1	Lung cancer	0.81	1.11
H322	Lung cancer	14.90	73.97
H1299	Lung cancer	7.67	4.57
MDA-MB-231	Breast carcinoma	0.78	1.11
MCF-7	Breast carcinoma	6.34	2.78
MDA-MB-468	Breast carcinoma	1.34	1.44
LoVo	Colon carcinoma	2.19	1.07
Colo205	Colon carcinoma	2.42	2.01
A2780	Ovarian cancer	4.60	27.3
SKOV3	Ovarian cancer	1.44	1.73
MKN45	Gastric cancer	18.19	16.06
PNAC-1	Pancreatic cancer	1.17	6.57
K562	Leukemia	2.87	4.32
OPM2	Multiple myeloma	0.23	0.56

^a^ Each value is the mean of at least three experiments.

#### In vivo antitumor activity assay

**Figure 2.**
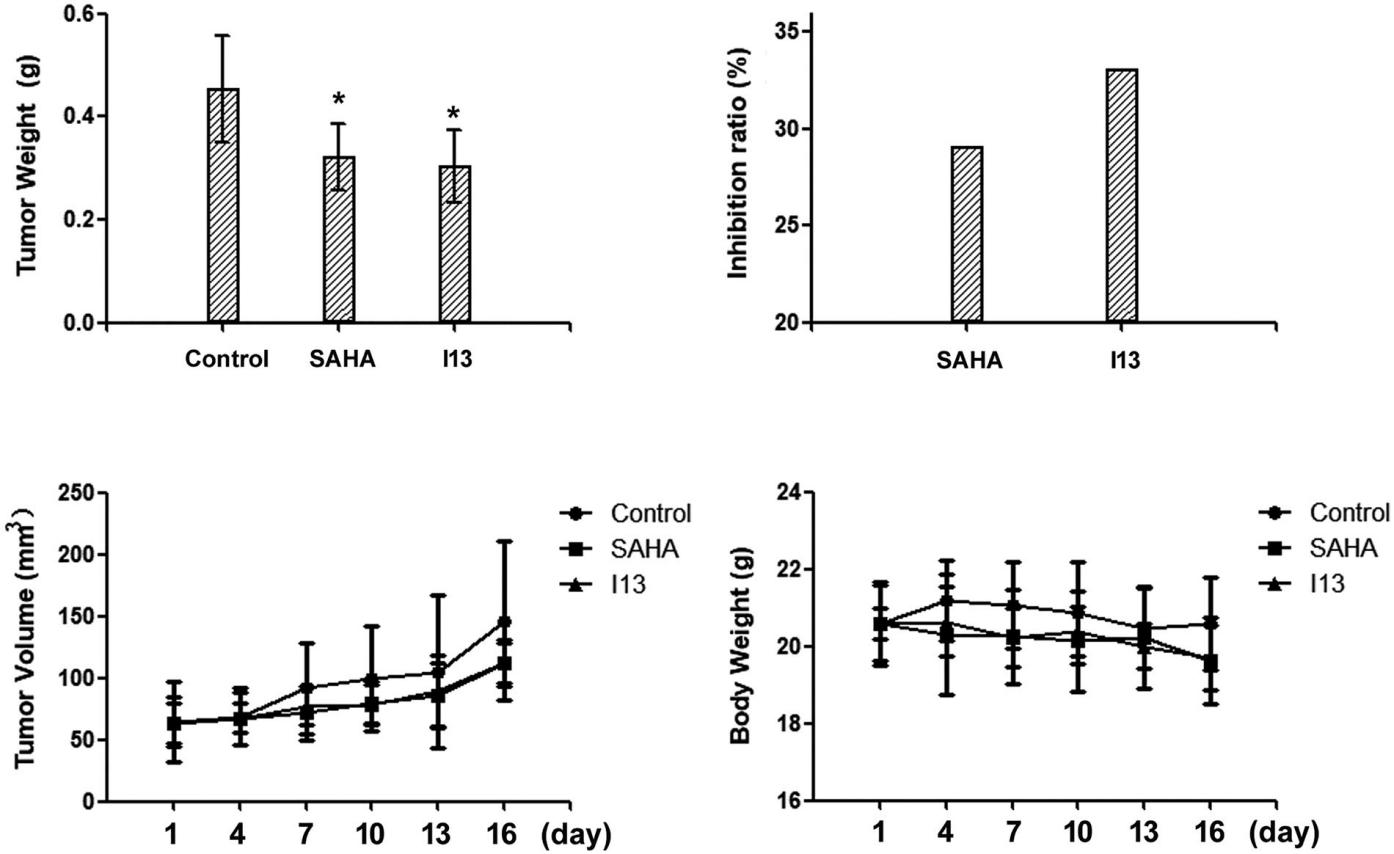
(a) Tumour weight plot of negative control group, positive control group (SAHA), and **I13** group; (b) inhibitory ratio plot of SAHA and **I13**; (c) tumour volume plot of these experimental groups (negative control, SAHA, and **I13**); (d) mice body weight plot of these experimental groups (negative control, SAHA, and **I13**). *Statistically different from control (*n* = 7).

**Figure 3.**
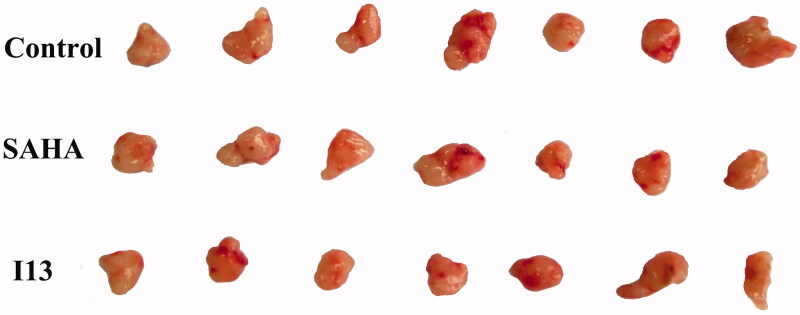
Illustration of dissected tumour tissues.

To further investigate anticancer potency of the derived molecules, molecule **I13** exhibited both good water solubility and high antiproliferative potency was selected for the *in vivo* anti-proliferation assay. Considering the tumourigenicity and sensitivity to the synthesised molecules, HepG2 cell line was selected for the inoculation of xenograft model. To preliminarily investigate the *in vivo* anti-proliferation ability of molecule **I13**, only one concentration (50 mg/kg/d) was utilised and SAHA (100 mg/kg/d) was used as a positive control. After 16 days of intragastric administration, the mice were executed, and the tumour weight was measured. The mean weight of tumours in the negative control group was 0.453 g (SD 0.10), and the values of SAHA groups and **I13** group were 0.322 g (SD 0.06) and 0.303 g (SD 0.07), respectively (Figure 2(a)). The inhibitory ratio of molecule **I13** was 33.1% compared with SAHA with a value of 29.0% (Figure 2(b)). The result showed that **I13** exhibited anti-tumour activity at dose of 50 mg/kg/d compared with a higher dose of SAHA (100 mg/kg/d). The tumour volume was measured every two days, and the result has correlation with the tumour weight (Figures 2(c) and 3). No evidence of mice body weight (measured every two days) loss indicated that molecule **I13** is safe (Figure 2(d)). Moreover, no obvious toxic signs in liver and spleen were detected.

#### Cell apoptosis assay

**Figure 4.**
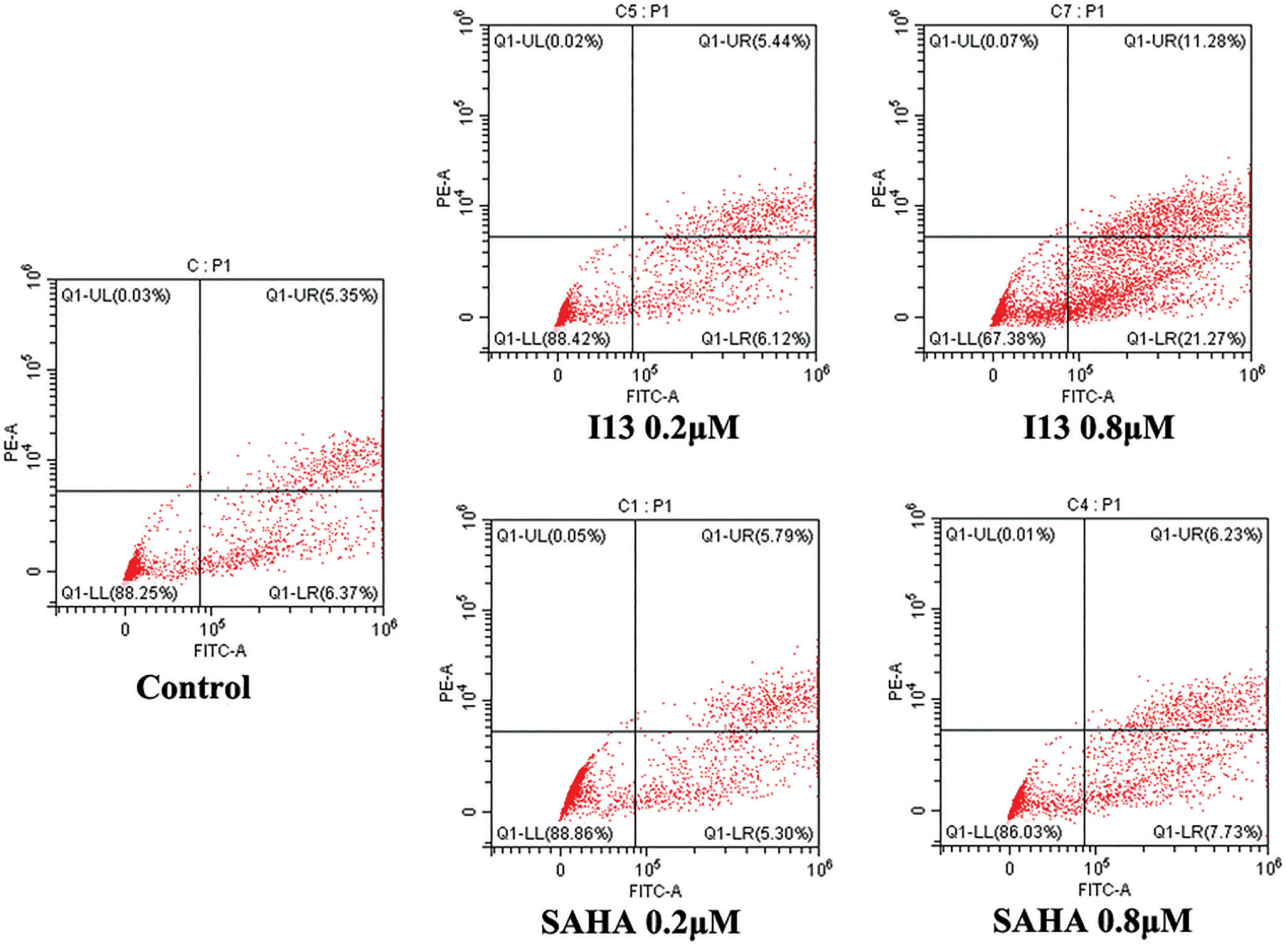
Pro-apoptotic effect of molecule **I13**.

Apoptosis which is a form of programmed cell death maintains the healthy survival/death balance in human cells. Increasing apoptosis rate could be utilised as a strategy for the treatment of cancer. To further evaluate the effect of **I13** on apoptosis, flow cytometry analysis was performed by staining HepG2 cells with annexin V-FITC/PI. The results revealed that both **I13** and SAHA treatment induced HepG2 cell apoptosis in a dose dependent manner (Figure 4). After treatment with difference doses of **I13**, the ratio of apoptotic cells was 11.56% and 33.55% at dose of 0.2 and 0.8 µM, respectively, compared with SAHA (11.09% and 13.96% at concentration of 0.2 and 0.8 µM, respectively). It is indicated that induction of cell apoptosis makes contributions to the antitumor effect of **I13**.

## Conclusions

Structural modification was performed on the previous IBA based HDACI. Compounds with hydroxamic acid and hydrazide groups as ZBGs were synthesised and evaluated in the enzyme inhibitory assay and *in vitro* antiproliferative screening. Among the derived compounds, molecule **I13** exhibited high HDAC inhibitory activity against HDAC1 (IC_50_ value of 13.9 nM), HDAC3 (IC_50_ value of 12.1 nM), and especially HDAC6 (IC_50_ value of 7.71 nM). The *in vitro* antiproliferative results revealed that molecule **I13** can effectively inhibit the growth of both haematologic and solid tumour cell lines. Compared with SAHA, molecule **I13** showed high potency in the growth inhibition of U937, U266, HepG2, A2780, and PNAC-1 cells. In the further *in vivo* assay, 50 mg/kg/d of **I13** can inhibit growth of xenograft tumour in athymic mice compared with 100 mg/kg/d of SAHA. Induction of apoptosis was revealed to play a role in the anticancer activity of molecule **I13**.The results suggested that HDACIs with high anticancer potency could be derived by further structural modification of the lead compound **I13**.
